# On the function of TRAP substrate-binding proteins: Conformational variation of the sialic acid binding protein SiaP

**DOI:** 10.1016/j.jbc.2024.107851

**Published:** 2024-09-30

**Authors:** Te-Rina J. King-Hudson, James S. Davies, Senwei Quan, Michael J. Currie, Zachary D. Tillett, Jack Copping, Santosh Panjikar, Rosmarie Friemann, Jane R. Allison, Rachel A. North, Renwick C.J. Dobson

**Affiliations:** 1 Biomolecular Interaction Centre, School of Biological Sciences, University of Canterbury, Christchurch, New Zealand; 2 Computational and Structural Biology Division, Victor Chang Cardiac Research Institute, Darlinghurst, New South Wales, Australia; 3 Biomolecular Interaction Centre, Maurice Wilkins Centre for Molecular Biodiscovery, and School of Biological Sciences, University of Auckland, Auckland, New Zealand; 4 Australian Synchrotron, ANSTO, Clayton, Victoria, Australia; 5 Department of Molecular Biology and Biochemistry, Monash University, Melbourne, Victoria, Australia; 6 Centre for Antibiotic Resistance Research (CARe) at University of Gothenburg, Gothenburg, Sweden; 7 School of Medical Sciences, Faculty of Medicine and Health, University of Sydney, Sydney, New South Wales, Australia; 8 Department of Biochemistry and Pharmacology, Bio21 Molecular Science and Biotechnology Institute, University of Melbourne, Parkville, Victoria, Australia

**Keywords:** membrane transport, membrane protein, tripartite ATP-independent periplasmic (TRAP) transporters, substrate binding proteins, sialic acid

## Abstract

Tripartite ATP-independent periplasmic (TRAP) transporters are analogous to ABC transporters in that they use a substrate-binding protein to scavenge metabolites (*e.g.*, *N*-acetylneuraminate) and deliver them to the membrane components for import. TRAP substrate-binding proteins are thought to bind the substrate using a two-state (open and closed) induced-fit mechanism. We solved the structure of the TRAP *N*-acetylneuraminate substrate-binding protein from *Aggregatibacter actinomycetemcomitans* (*Aa*SiaP) in both the open ligand-free and closed liganded conformations. Surprisingly, we also observed an intermediate conformation, where *Aa*SiaP is mostly closed and is bound to a non-cognate ligand, acetate, which hints at how *N*-acetylneuraminate binding stabilizes a fully closed state. *Aa*SiaP preferentially binds *N*-acetylneuraminate (*K*_D_ = 0.4 μM) compared to *N*-glycolylneuraminate (*K*_D_ = 4.4 μM), which is explained by the closed-*N*-acetylneuraminate bound structure. Small-angle X-ray scattering data alongside molecular dynamics simulations suggest the *Aa*SiaP adopts a more open state in solution than in a crystal. However, the open unliganded conformation can also sample closed conformations. Molecular dynamics simulations also demonstrate the importance of water molecules for stabilizing the closed conformation. Although our data is consistent with an induced fit model of binding, we suggest that the open unliganded conformation may sample multiple states capable of binding substrate. The mechanism by which the ligand is released for import remains to be determined.

Sialic acids are an abundant family of nine-carbon sugars with many functions in mammals, including cell-cell recognition and signaling. More than 50 variants of sialic acid are known, but the most common in humans is *N*-acetylneuraminate (Neu5Ac). Sialic acids are scavenged by pathogenic and commensal bacteria as a source of energy or to evade the human immune system (1, 2). To utilize host-derived sialic acids, however, most bacteria must first import them using dedicated sialic acid transporters [(3) and reviewed in (4, 5)]. Disruption of bacterial sialic acid transporters (*i.e.*, knockouts) impairs the growth, survival, and colonization of pathogenic bacteria (2, 6, 7, 8, 9), highlighting the potential of these systems as antibacterial drug targets. Upon internalization, Neu5Ac is converted to *N-*acetylmannosamine, *N-*acetylmannosamine-6-phosphate, and *N-*acetylglucosamine-6-phosphate by the enzymes *N*-acetylneuraminate lyase (*nan*A), *N*-acetylneuraminate kinase (*nan*K), and *N*-acetylmannosamine-6-phosphate 2-epimerase (*nan*E) (10, 11, 12, 13, 14). Of particular interest are the sialic acid transporters of the tripartite ATP-independent periplasmic (TRAP) transporter family, as they are only present in bacteria and archaea (15) so could be targeted without affecting the human host.

TRAP transporters are secondary active transporters that transport specific ligands across bacterial membranes by co-transporting counter ions down an electrochemical gradient [(15, 16, 17, 18, 19, 20) and recently reviewed here (21)]. Analogous to the ATP-binding cassette (ABC) transporter superfamily, TRAP transporters utilize a high-affinity substrate-binding protein that binds a target ligand with high affinity and delivers it to the membrane-spanning components for uptake (22, 23). In the sialic acid-specific TRAP system (SiaPQM), the substrate-binding protein, SiaP, is required for transport (18, 19, 21, 24) and associates with the membrane-spanning subunit, SiaQM, to facilitate the translocation of sialic acid through an elevator-type transport mechanism.

Currently, 23 high-resolution structures are available for SiaP proteins (including various mutants) from just five species of Gram-negative bacteria: *Haemophilus influenzae*, *Vibrio cholerae*, *Fusobacterium nucleatum*, *Pasteurella multocida*, and *Photobacterium profundum* (listed in Table S1). This wealth of structural data has provided insights into the molecular basis for the high affinity and specificity of SiaP proteins for sialic acids, particularly Neu5Ac and *N*-glycolylneuraminate (Neu5Gc) (25, 26, 27, 28). The structural core of these substrate-binding proteins consists of two globular domains connected by a variable hinge region (29), which is formed by two β-strands and a unique extended α-helix in SiaP. Ligands bind within the cleft between the two domains, which close around the bound ligand *via* a hinge-bending conformational change often described as a “Venus flytrap” mechanism (30, 31). The available crystal structures for SiaP without ligand adopt open conformations, suggesting that closure is strictly ligand-induced, consistent with a two-state induced fit binding model (27).

It remains unclear to what extent unbound SiaP undergoes intrinsic closure in solution like other substrate-binding proteins (32, 33, 34, 35). SiaP conformational dynamics have been investigated using pulsed electron-electron double resonance spectroscopy and single-molecule FRET, which support the proposal that *V. cholerae* SiaP (*Vc*SiaP) exclusively occupies an open conformation in the absence of ligand and that ligand binding induces and stabilizes closure (36, 37). Molecular dynamics simulations uniquely developed to reproduce the DEER signal from these experiments suggest that *Vc*SiaP generally adopts a more open conformation in solution than the observed from the crystal structure, and is conformationally flexible, but does not fully close without the ligand (38). Together, these data present a sensible model for SiaP dynamics, although it is unclear whether this model can be applied to all SiaP proteins, or whether there is variation between homologs. Furthermore, the precise details of how sialic acid binding may trigger the conformational change in SiaP are yet to be elucidated.

Here, we report the functional, biophysical, and structural characterization of the substrate-binding protein of the sialic acid TRAP transporter from *Aggregatibacter actinomycetemcomitans* (*Aa*SiaP), a known periodontal pathogen that can also cause infective endocarditis (39, 40). We demonstrate that *Aa*SiaP preferentially binds Neu5Ac over Neu5Gc with micromolar affinity and that sodium ions are not involved in binding. Our findings suggest that *Aa*SiaP can adopt a mostly closed conformation that is stabilized by acetate, a small non-cognate ligand. Molecular dynamics simulations and small-angle X-ray scattering data support the notion that *Aa*SiaP has greater conformational flexibility than is evident from the crystal structures.

## Results

### Bioinformatic analysis demonstrates that *Aa*SiaP shares key residues for Neu5Ac binding

**Figure 1:**
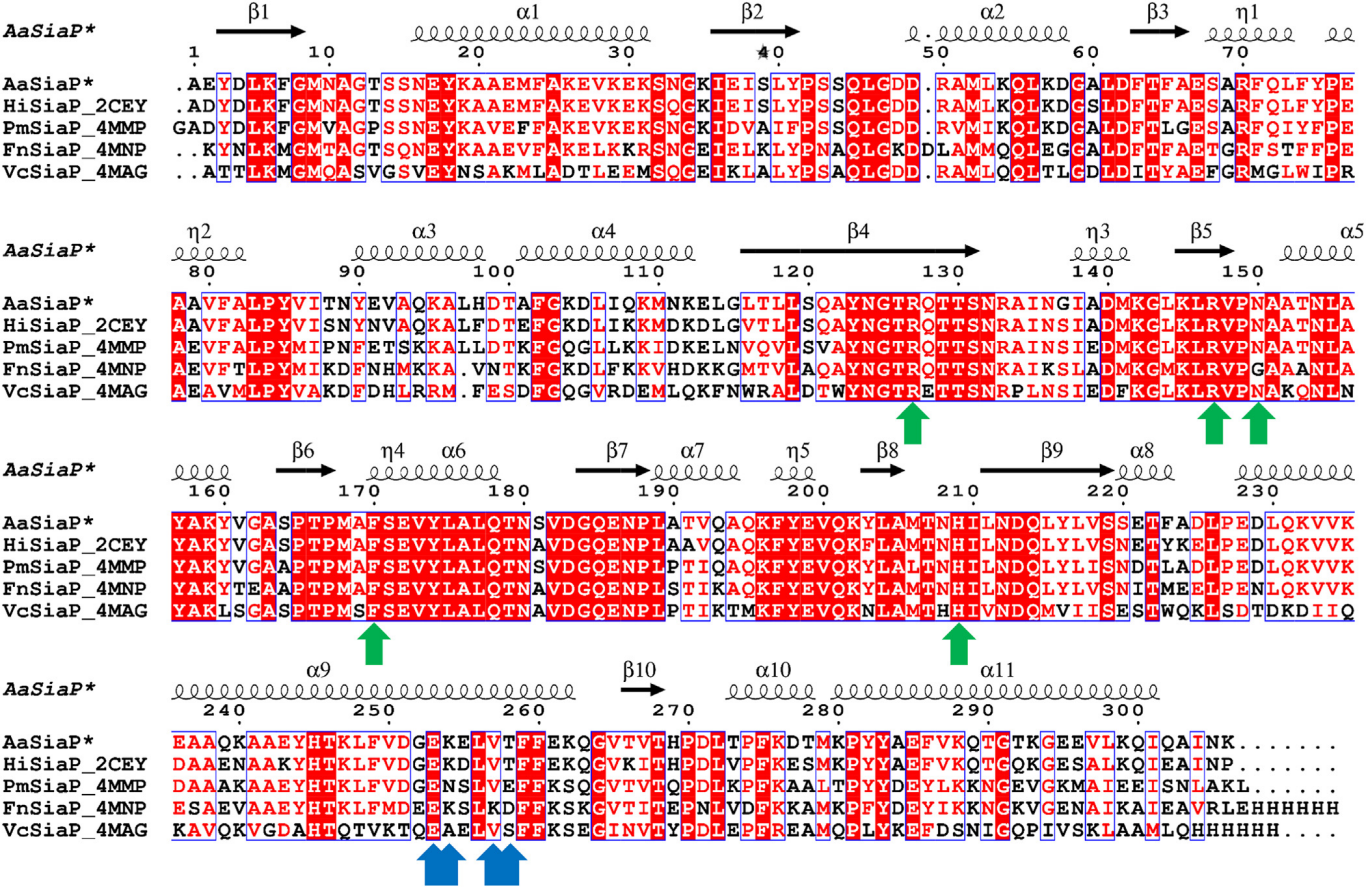
**Multiple sequence alignment of Neu5Ac-binding proteins homologous to *Aa*SiaP with structures deposited in the PDB.** The residue numbering and secondary structure is based on *Hi*SiaP. Sequence identity between *Aa*SiaP and each homolog is as follows: *Hi*SiaP 87.5%, *Pm*SiaP 74.9%, *Fn*SiaP 66.5%, and *Vc*SiaP 51.6%. *Red background* indicates complete sequence identity and *red letters* indicate residues with similar physicochemical properties. ∗Recombinant *Aa*SiaP used in this study has an N-terminal cleavage fragment comprising an M and D residue scar not shown here. Created in ESPript 3.0 (https://espript.ibcp.fr/ESPript/cgi-bin/ESPript.cgi). A broader sequence alignment of Neu5Ac-binding proteins is in Figure S1. Residues involved in Neu5Ac binding are marked with a *green arrow*. Residues in the hinge helix α9 where the bend/kink forms upon Neu5Ac binding are marked by *blue arrows*.

We first conducted a bioinformatic analysis of the putative *siaP* gene from *A. actinomycetemcomitans* (*Aa*SiaP). To verify that *siaP* is correctly annotated in the *A. actinomycetemcomitans* genome, a basic local alignment search tool (BLAST) analysis was performed with the *Aa*SiaP amino acid sequence. The *Aa*SiaP sequence showed a high level of similarity to the amino acid sequences of confirmed sialic acid substrate-binding proteins (SiaP), consistent with its proposed role in sialic acid metabolism. A sequence alignment (Fig. 1) demonstrates that many of the key residues for sialic acid binding and specificity [including R127, R147, F170, and H209 (26, 28)] are conserved in the *Aa*SiaP primary structure, supporting its identity as a sialic acid substrate-binding protein. Interestingly, the well-conserved surface residue N150, which could be important for transport, was also present in *Aa*SiaP and 11 of 12 homologs but substituted to glycine in *Fn*SiaP. The bioinformatic survey provided 12 sequences of homologous proteins (50–95% sequence identity, Fig. S1), with four of these structurally characterized using X-ray crystallography, which provides potential templates for molecular replacement of *Aa*SiaP (Fig. 1).

### *Aa*SiaP demonstrates a preference for *N*-acetylneuraminate over *N*-glycolylneuraminate

*Aa*SiaP was recombinantly produced in *Escherichia coli* for subsequent structural and biophysical analyses. To facilitate the folding of the protein in the *E. coli* periplasm, the native *A. actinomycetemcomitans* periplasmic signal sequence was replaced with the *pelB* periplasmic signal sequence. Recombinant *Aa*SiaP was highly produced and could be isolated from the periplasmic fraction (Fig. S2*A*) and purified by anion-exchange, hydrophobic-interaction, and size-exclusion chromatography (Fig. S2*B*). *Aa*SiaP eluted from the size-exclusion column as a single peak (Fig. S2*C*), suggesting that it is a monodisperse species and remains stable throughout purification.

To confirm the function of *Aa*SiaP we used two solution-based techniques that detect temperature-related changes in fluorescent intensity to estimate the affinity and thermal stabilization of ligand binding (Table 1).

**Table 1:** Estimated dissociation constant (*K*_D_) values for *Aa*SiaP binding to Neu5Ac and Neu5Gc from microscale thermophoresis and thermal stability of Neu5Ac binding from differential scanning fluorimetry in the presence of either K^+^ or Na^+^

Dissociation constant (95% CI)^a^	
Ligand	*K*_D_*Aa*SiaP-FITC (nM)
Neu5Ac	430 (136–724)
Neu5Gc	4400 (2600–6200)


Thermal stability ± SD^b^		
Salt	*Aa*SiaP T_m_ (°C)	T_m_ + 50 μM Neu5Ac (ΔT_m_) (°C)
NaCl	50.7 ± 0.6	57.2 ± 0.9 (+6.4)
KCl	51.1 ± 0.3	58.6 ± 0.6 (+7.5)

a Determined by microscale thermophoresis.

b Determined by differential scanning fluorimetry.

**Figure 2:**
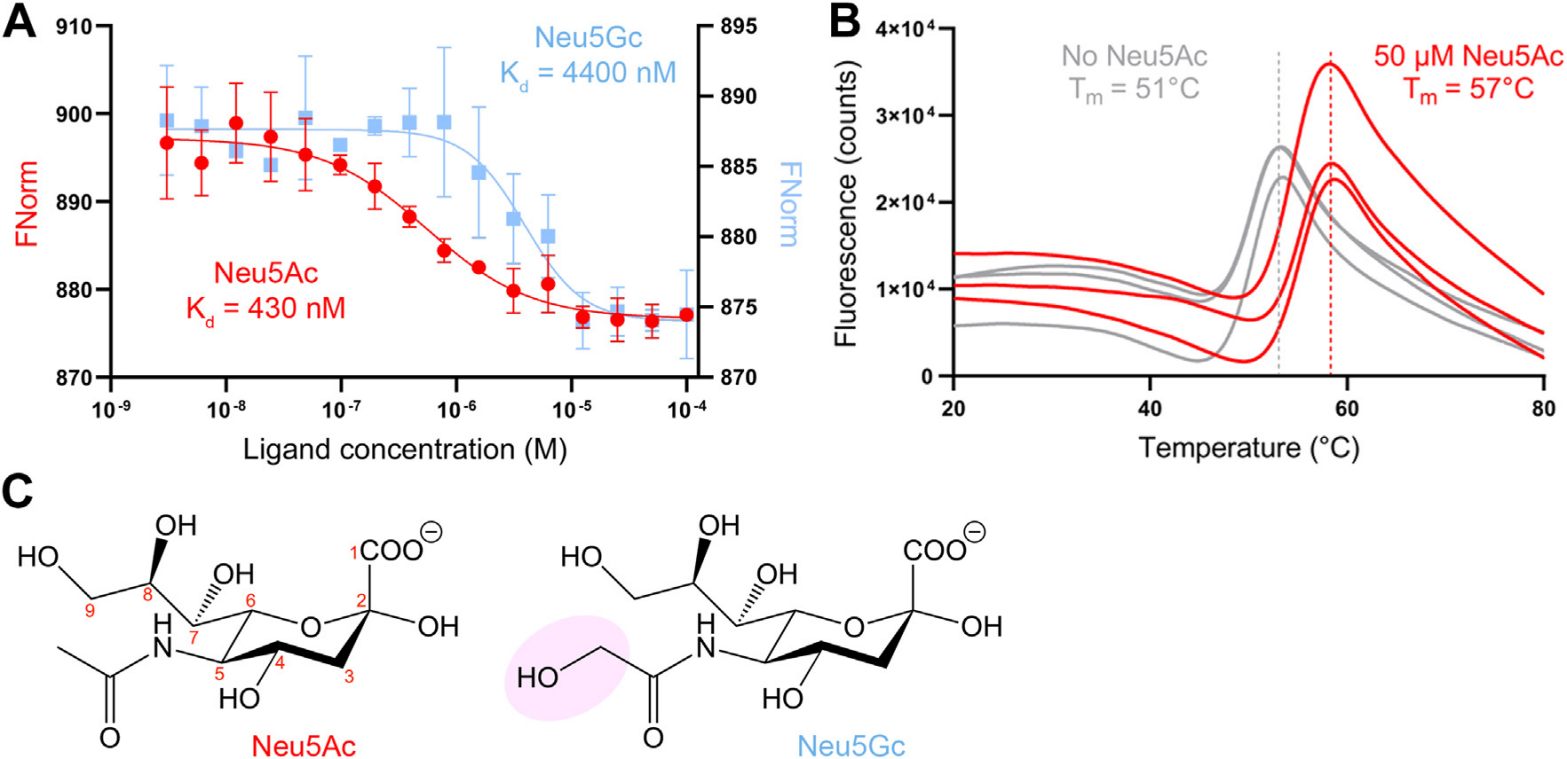
**Ligand binding to *Aa*SiaP.***A*, *Aa*SiaP (75 nM) binding curve and affinity (*K*_D_) for Neu5Ac (*red circle*) and Neu5Gc (*light blue squares*) determined by microscale thermophoresis, errors bars indicate standard deviation. *B*, thermal shift assay results for *Aa*SiaP (1 μM) showing an increase in melting temperature (T_m_) from apo (*grey curves*) with the addition of 50 μM Neu5Ac (*red curve*), determined by differential scanning fluorimetry, individual replicates are shown. *C*, the structures of Neu5Ac and Neu5Gc, highlighting their difference (*shaded pink*).

Microscale thermophoresis was used to estimate the binding affinity of *Aa*SiaP to the sialic acids *N*-acetylneuraminate (Neu5Ac) and *N*-glycolylneuraminate (Neu5Gc) (Fig. 2*A* and Table 1). The protein was labelled with fluorescein-5-isothiocyanate (FITC) directed towards the N-terminal free amine (41) located in the less dynamic domain I to minimze potential fluorophore-protein interactions. However, this conservative labelling approach gave a low fluorophore/protein molar ratio and some fluorescent counts were below the recommended threshold due to rapid photobleaching (Fig. S3, *B*–*D*). Despite this, we obtained estimates of *Aa*SiaP binding affinity (*K*_D_) of 430 nM for Neu5Ac (95% CI: 136–724 nM) and 4400 nM for Neu5Gc (95% CI: 2600–6200 nM) that are in good agreement with published values for SiaP homologues from *H. influenzae*, *P. multocida*, and *F. nucleatum* (Table S1).

Neu5Ac binding was also estimated using differential scanning fluorimetry thermal shift assays with unlabeled *Aa*SiaP (Fig. 2*B* and Table 1). This experiment demonstrates that *Aa*SiaP is stable at room temperature (T_m_ with Tris-NaCl = 50.7 ± 0.6 °C) and is further stabilized by Neu5Ac binding (T_m_ with 50 μM Neu5Ac = 57.2 ± 0.9 °C [an increase of 6.4 °C]) to a similar extent as *Hi*SiaP (25). TRAP transporters use Na^+^ ion gradients to drive transport (17, 19, 21, 24) and one intriguing possibility is that the Na^+^ ions are also bound and delivered to the transporter by the substrate-binding protein. *Aa*SiaP was similarly stabilized by Neu5Ac in the absence of Na^+^ ions (T_m_ with Tris-KCl + 50 μM Neu5Ac = 58.6 ± 0.6 °C [an increase of 7.5 °C]). Titration of Neu5Ac into *Aa*SiaP and monitoring for a change in melting temperature estimated the *K*_D_ to be 54.6 μM (95% CI: 21–122 μM) when in Tris-NaCl buffer, and this is largely unchanged in the absence of Na^+^ ions (Tris-KCl buffer), where the *K*_D_ is estimated to be 37.9 μM (95% CI: 23–62 μM) (Fig. S3*A*). The discrepancy in the *K*_D_ between the MST and DSF experiments is expected because in DSF the temperature is increased.

Together, microscale thermophoresis and thermal shift assays demonstrate that *Aa*SiaP binds both Neu5Ac and Neu5Gc, but with a clear preference for Neu5Ac. Sodium ions (used by TRAP transporters for symport) do not appear to be directly involved in the binding of Neu5Ac to *Aa*SiaP—consistent with available crystal structures that do not show any obvious sodium binding sites.

### The crystal structures of unbound- and Neu5Ac-bound *Aa*SiaP reveal a series of distinct conformations

To better define the molecular mechanism by which *Aa*SiaP binds Neu5Ac and to complement our solution data, we determined the crystal structure of *Aa*SiaP in both the absence and presence of Neu5Ac. Of the 23 SiaP structures deposited in the Protein Data Bank (PDB), 16 are variations of *Hi*SiaP (residue substitutions, different ligands) and another four are variations of *Vc*SiaP (Table S2), so there is a need to study further homologs to understand the generality of the binding mechanism. Moreover, there are just two structures of SiaP in the open conformation (*i.e.*, unbound-SiaP), limiting a generalized understanding of how these proteins bind Neu5Ac with high affinity.

**Figure 3:**
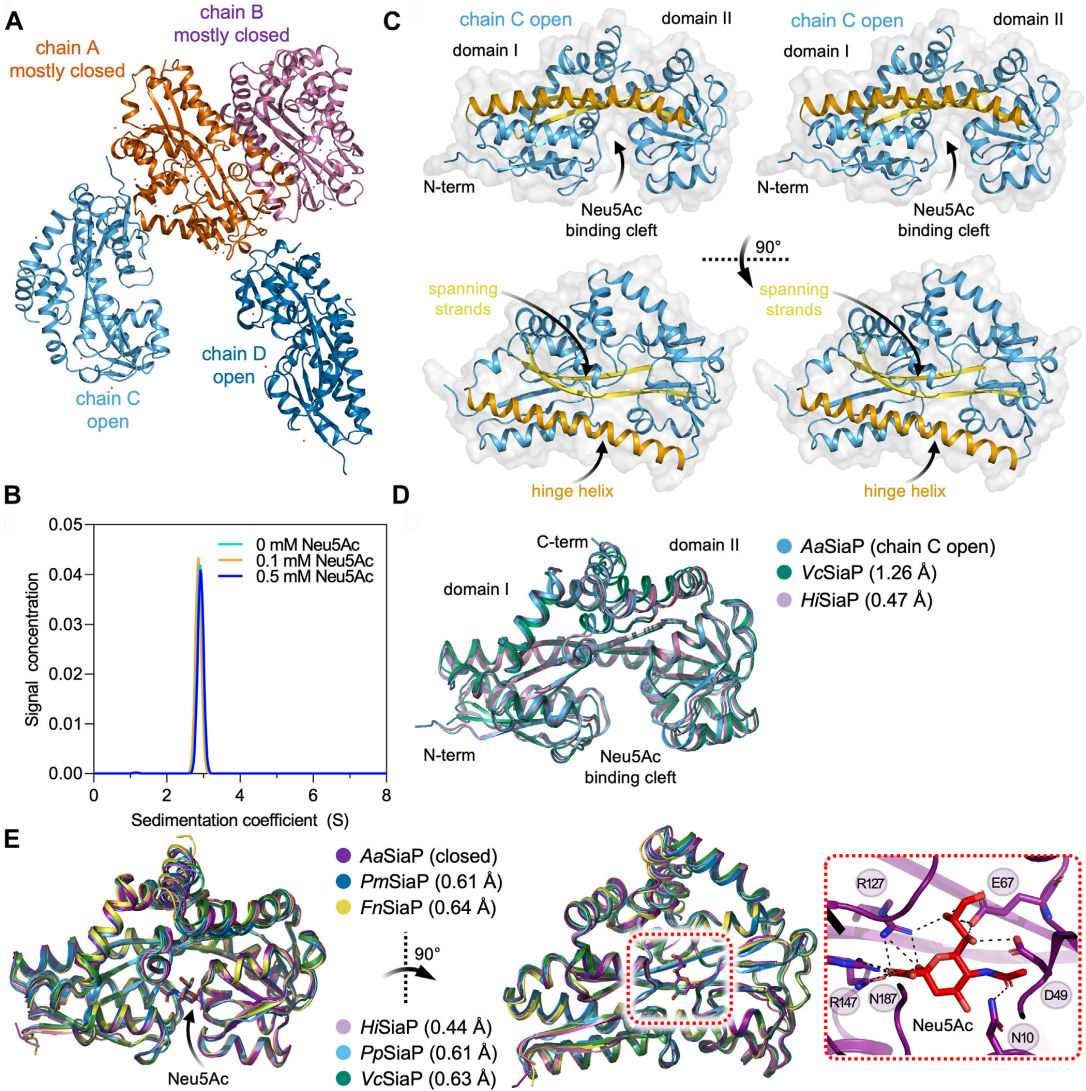
**Crystal structure of *Aa*SiaP.***A*, asymmetric unit of ligand-free *Aa*SiaP contains four monomers. Chains *A* and *B* are in an acetate-bound conformation, whereas chains *C* and *D* are in an open conformation. PISA analysis (https://www.ebi.ac.uk/pdbe/pisa/) demonstrates that the largest interface is between chains *A* and *B* (∼530 Å^2^). *B*, sedimentation velocity AUC analysis of *Aa*SiaP at various concentrations of Neu5Ac shows a well-defined single peak at ∼2.8 S. This is indicative of a monodisperse and monomeric protein in solution that is not affected by the presence of Neu5Ac. Fits to the data are in Figure S4. *C*, stereo plots (cross-eyed) showing the overall architecture of *Aa*SiaP (chain *C*). *D*, an overlay of homologous SiaP structures in the equivalent open conformation [*Vc*SiaP (4MAG (28)) and *Hi*SiaP (2CEY (27))] shows that the structure is highly conserved. *E*, an overlay of homologous SiaP structures in the equivalent ligand-bound, closed conformation [*Pm*SiaP (4MMP (28)), *Fn*SiaP (4MNP (28)), *Hi*SiaP (6H76 (25)), *Pp*SiaP (7T3E (17)), and *Vc*SiaP (7A5Q (37))]. In all structures, Neu5Ac is bound in the same pose. The inset shows the *Aa*SiaP residues binding Neu5Ac.

Unbound-*Aa*SiaP easily afforded crystals in a variety of conditions, the best of which diffracted to a maximum resolution of 2.45 Å. Despite having very good search models, we initially struggled to solve the phases by molecular replacement for the unbound-*Aa*SiaP structure. The Matthew’s coefficient estimated four to six molecules in the asymmetric unit, but molecular replacement phasing attempts using an unliganded *Hi*SiaP structure in the open conformation as the search model (88% sequence identity, PDB ID: 2CEY) placed only two molecules. Attempts to phase with Neu5Ac-bound *Pm*SiaP in the closed conformation (75% sequence identity, PDB ID: 4MMP) also only yielded a partial solution of two molecules. A successful solution was found by searching for two molecules of *Hi*SiaP structure in the open conformation and two *Pm*SiaP monomers in a closed conformation, despite no Neu5Ac being added during purification or crystallization. Thus, the crystal structure revealed that unbound-*Aa*SiaP has two distinct conformations present as different chains in the asymmetric unit (Fig. 3*A*). One conformer is clearly in an open conformation, without a bound ligand. The other conformer we refer to as “mostly closed” is bound to what we model as an acetate ion (the crystallization condition contained 200 mM acetate). The final refined model had an *R*_free_ of 24.6% and a *R*_work_ of 18.5%, with reasonable geometry and Ramachandran outliers for the resolution (2.45 Å), as judged by MolProbity (42, 43) (Table 2).

**Table 2:** Crystallographic data collection and refinement statistics for recombinant *Aa*SiaP

	Ligand free-*Aa*SiaP	Neu5Ac-bound *Aa*SiaP
Data collection		
Temperature (K)	100	100
Detector	EIGER X 16M (dectris)	EIGER X 16M (dectris)
Crystal-to-detector distance (mm)	300	200
Space group	*C2*	*P 2*_*1*_
Unit cell parameters [a, b, c (Å); β (°)]	138.7, 141.4, 109.4; 112.5	66.2, 49.5, 121.4; 92.8
Resolution range (Å)	48.64–2.58 (2.65–2.58)	45.86–1.90 (2.0–1.90)
No. of unique reflections	61,226 (2476)	62,386 (6175)
Multiplicity (outer shell)	3.6 (3.7)	2.0 (2.0)
Completeness (%) (outer shell)	100.0 (99.9)	99.76 (99.72)
*I*/σ(*I*) (outer shell)	6.3 (1.6)	6.2 (1.18)
CC_(1/2)_ (outer shell)	0.995 (0.783)	0.995 (0.505)
*R*_merge_ (outer shell)	0.087 (0.612)	0.064 (0.641)
Refinement statistics		
No. of reflections used	58,058	62,286
No. of protein atoms	9609	4862
No. of water molecules	49	487
*R*_work_ (%)	18.9	20.0
*R*_free_ (%)	22.0	23.1
Reflections used for *R*_free_	3158 (243)	3107 (127)
Ramachandran (% favoured/allowed/disallowed)	99.6, 0.3, 0.08	98.4, 1.6, 0
r.m.s.d. bond lengths (Å)	0.015	0.014
r.m.s.d. angles (°)	1.58	1.461
Clashscore (*Phenix*)	4.4	3.16
PDB id	9BH3	9BHF

We also solved a 1.9 Å crystal structure of Neu5Ac-bound *Aa*SiaP. Molecular replacement was straightforward in this case using a liganded *Hi*SiaP structure (PDB ID: 2WYK). The structure closely resembles the Neu5Ac-bound *Hi*SiaP structure (root mean square difference (r.m.s.d.) = 0.44 Å, across 283 C_α_-atoms) and can be described as a fully closed, bound conformation. For both models, refinement was initially performed with simulated annealing to remove model bias. The observation that of the 23 SiaP structures deposited in the PDB, just two structures are in the open, ligand-free conformation (4MAG and 2CEY, Table S2) may reflect some difficulty in crystallizing SiaP proteins in the absence of Neu5Ac due to conformational flexibility. Anecdotally, we found *Aa*SiaP more easily crystallized in the presence of Neu5Ac, and the resolution is considerably better, which is consistent with the large stabilizing effect of Neu5Ac (T_m_ + 50 μM Neu5Ac = 57.2 ± 0.9 °C, an increase of 6.4 °C).

The monomeric structure of *Aa*SiaP is similar to other reported SiaP structures (see references in Table S2), first described for *Hi*SiaP (27). The structure has two domains that comprise residues from both the N-terminal and the C-terminal ends of the sequence (Fig. 3*C*). The domains are bridged by two long β-strands (spanning strands, β3 and β4) and a long α-helix (the hinge helix, α9) that together are known as the ‘hinge region’, and form a cleft within which Neu5Ac binds.

Comparing the open conformation with equivalent homologs in the PDB shows that the open structure is highly conserved (Fig. 3*D*), *Aa*SiaP to *Vc*SiaP (4MAG) r.m.s.d. = ∼1.23 Å across 278 C_α_-atoms and *Aa*SiaP to *Hi*SiaP (2CEY) r.m.s.d. = 0.5 Å across 279 C_α_-atoms (27, 28) (Table S3). The closed Neu5Ac-bound structure is also highly conserved when compared with equivalent homologs (r.m.s.d. = 0.4–0.6 Å across five homologues, Fig. 3*E*). In all available cases, the binding pose for Neu5Ac is identical and the residues that bind Neu5Ac are well conserved (Fig. S5*B*). Recent work has highlighted the importance of water networks for Neu5Ac binding (25) and again we find that the water network around Neu5Ac is also highly conserved (Fig. S5, inset outlined in blue).

PISA analysis (44) predicts that the most significant interface is between the mostly closed monomers (chains A and B, Fig. 3*A*) in the ligand free-*Aa*SiaP crystal (∼530 Å^2^). To check whether *Aa*SiaP can oligomerize we conducted analytical ultracentrifugation sedimentation velocity experiments in the presence and absence of Neu5Ac (Fig. 3*B*). In all cases, the samples present a single symmetrical peak at ∼2.9 S. The estimated mass of *Aa*SiaP is 34 to 35 kDa based on these experiments, which is consistent with the mass calculated from the amino acid sequence (34.3 kDa). No detectable change in the frictional ratio (a measure of asymmetry) was found when Neu5Ac was added (*f*/*f*_o_ = 1.27–1.28), suggesting that sedimentation velocity experiments are not sensitive enough to detect the change in shape.

### Conformational change upon Neu5Ac binding

**Figure 4:**
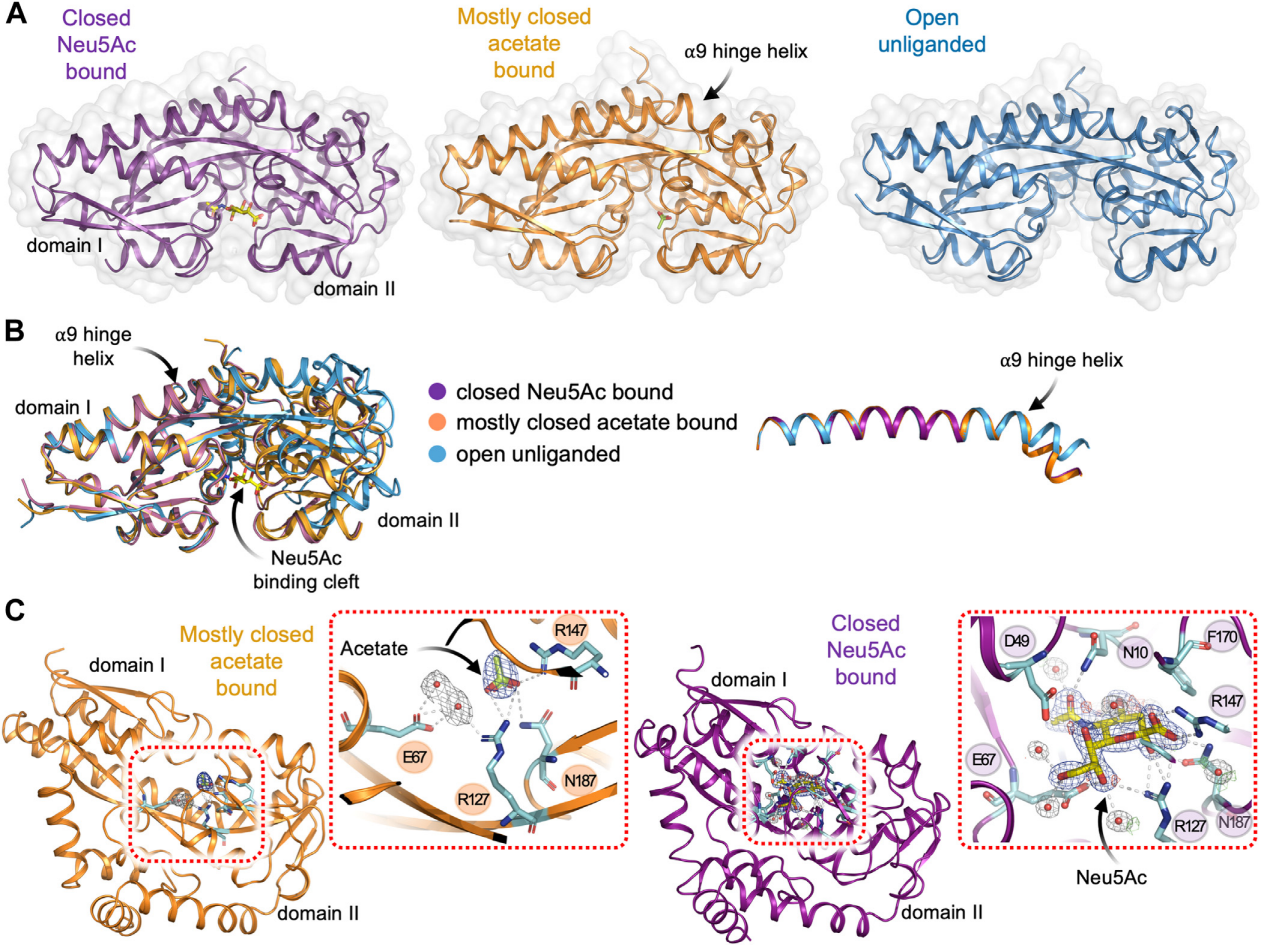
**The conformational landscape of *Aa*SiaP.***A*, in *purple*, the closed Neu5Ac-bound conformation, showing Neu5Ac bound deep within a cleft between the N-terminal (domain I) and C-terminal (domain II) lobes. In *orange* is the acetate-bound conformation, where acetate is also bound by twin conserved arginine residues of domain II. In *blue*, the open unliganded conformer from the same crystal structure as the acetate-bound conformer. As depicted, the α9 hinge helix running between the domains undergoes a substantial hinge bending motion to allow the two domains to come together. *B*, an overlay of the three conformations of *Aa*SiaP reported here. The bend in the α9 hinge helix is highlighted in the *right pane*. *C*, zoom-in of the substrate-binding site, highlighting ordered water molecules and hydrogen bonding interactions (*white dashes*). Electron density (2mF_o_-DF_c_ contoured at 1*σ*, mF_o_-F_c_ contoured at 3*σ*) at the substrate-binding site of the acetate-bound structure (*left*) and closed Neu5Ac-bound structure (*right*).

We compared the three structures of *Aa*SiaP to define the motions the protein undergoes in solution and when binding Neu5Ac. As previously described, the conformational changes associated with ligand-binding of *Aa*SiaP can be thought of as essentially a rigid body domain movement about a hinge region. The rotation angle is 23° when analyzed using DynDom, which is in line with the previous analysis of *Hi*SiaP (25–31°) (27). An overlay of the open and mostly closed conformations of unbound-*Aa*SiaP (Fig. 4, *A* and *B*) differed by an r.m.s.d. of ∼1.5 Å across 283 C_α_-atoms (Table S2). The two acetate-bound conformations more closely resemble the Neu5Ac-bound *Aa*SiaP (chain A r.m.s.d. = 0.4 Å, chain B = 0.3 Å) and *Hi*SiaP structures (r.m.s.d. = ∼0.4 Å; Table S2). The mostly closed acetate-bound structure represents a unique intermediate conformation that, to our knowledge, has not been reported for TRAP substrate-binding proteins but has been observed in ABC substrate-binding proteins (32, 35).

The closed conformation of TRAP substrate-binding proteins has only been observed in the presence of substrate, so we checked the mostly closed structure for a bound ligand. An electron density peak larger than that of a water molecule was evident near the highly conserved R147 and R127 residues that would otherwise coordinate the carboxyl group of Neu5Ac (Fig. 4*C*). This density is consistent with acetate, a small organic anion with a carboxyl moiety that was present in both crystallization conditions. Acetate makes favorable interactions with residues R127, R147, and N189 on domain II within the binding site of *Aa*SiaP, effectively mimicking the carboxyl moiety of Neu5Ac. However, unlike Neu5Ac, acetate does not bridge the two domains, which fails to explain the closure of the domains. We note that in chain B of the acetate-bound structure, acetate also makes contact with a chain of water molecules (Fig. 4*C*), which in turn contacts E67 in domain I, a residue that normally interacts with Neu5Ac. The resolution of the ligand free-*Aa*SiaP structure (2.58 Å) is lower compared to that of the Neu5Ac structure (1.9 Å), resulting in fewer evident waters. Furthermore, neither the acetate-bound nor open structures exhibit the shell of highly ordered and conserved waters observed in the Neu5Ac binding site when Neu5Ac is present. It remains unclear whether acetate binding induces a partial closure of *Aa*SiaP. However, if it does, this would suggest that the twin-arginine residues to which acetate binds, and perhaps also E67 from domain I, are involved in sensing the substrate and potentially play a role in triggering or stabilizing the conformational change towards partial closure, as originally proposed by (28).

The change from the open to the closed conformation in *Aa*SiaP induces a significant kink in the hinge helix α9 (Fig. 4*B*). Compared to the minor bend in hinge helix α9 when in the open conformation, the closure of the two domains around the Neu5Ac breaks two mainchain hydrogen bonds between E253-V257 and K254-T258 (∼5.4 Å between the heavy atoms) within the helix that allows it to kink. The packing of the hinge helix α9 sidechains against the two domains does not appreciably change as the domains close. This helix has been proposed to function as an energetic barrier and “switch” that holds SiaP in the closed conformation upon Neu5Ac binding (27). Aligning Neu5Ac bound *Aa*SiaP with other Neu5Ac bound SiaP homologues demonstrates that the bend occurs at the same position and the same hydrogen bonds within the helix are broken, suggesting this is a conserved mechanism. It is straightened completely in structures generated by molecular dynamics (MD) simulations (discussed in detail later).

The prime candidate for signaling the hinge region to open or close is the highly conserved triad of interacting residues R127, E186, and H209 (28), although this has been questioned in the literature (36). In the ligand-free state, R127 within the hinge region is proposed to form a strong interaction with E186, which is significantly weakened upon Neu5Ac binding and coordination by R127. This results in a stronger interaction between E186 and H209, which is also housed in the β-strand spanning area. This network has been implicated in triggering the observed conformational change, and H209 has also been implicated in the protein folding process (27). From a structural superposition of our crystal structures in different conformations, there do not appear to be any major changes in this network to support the proposed role in triggering the conformational change. However, in fully open structures obtained from relaxed MD simulations (next section) we observe that the interaction between E186 and H209 is weakened—the distance between the oxygen (OE2) of E186 and the nitrogen (NE2) of H209 increases from 2.7 Å to 4.0 Å. Together with our mostly closed structure with acetate bound at R127, we suggest that this residue interaction network is partly involved in stabilizing the open and closed conformations of SiaP.

Superposition of the structures also reveals that the largest movements upon closure occur at surface residues near the substrate-binding site, particularly R50, N150, F170, and the residues that they interact with. In the closed-bound structure, R50 and N150 are conspicuously stacked together at the protein surface, forming one of the few new residue-residue contacts between the N- and C-terminal domains upon closure (as analyzed by DynDom). We collectively refer to this entire region as a latch, which was also previously identified in the *H. influenzae* SiaP (45) and in the *P. profundum* SiaP (17). R50 undergoes the largest movement between the acetate-bound, unbound, and closed-bound states (∼5 Å). Indeed, mutation of the equivalent latch residues (R49 and N148) in *Pp*SiaP showed a dramatic reduction in transport activity using the full SiaP-SiaQM system (17). The neighboring residue D49 is also of interest here, as it coordinates both the glycerol moiety of Neu5Ac and R70. This, in turn, holds R70 in close contact with N150 bridging across the binding cleft, with the guanidinium group of R70 hydrogen bonding to the N150 backbone carbonyl. Of the new residue-residue contacts upon closure, the R70-N150 contact is one of two H-bonding interactions formed between domains, the other being Q72-T153. Both interactions are observed in the acetate-bound, unbound, and liganded structures, however, in the acetate-bound structure, the aforementioned R50-N150 stacking interaction is not present. Therefore, it appears that D49 is also involved in ligand sensing and protein closure, by stabilizing the interaction between R70 (domain I) and N150 (domain II). As previously mentioned, 11 of 12 SiaP homologs have the R50-N150 latch, and those that have structures available (3B50, 4MMP, 7T3E, and 7A5Q) have near identical positioning of the latch residues, as well as the ordered water molecules linking the substrate to the latch region. We note that in *Fn*SiaP (4MNP), the latch residues are L52-G151, which although make close contact in the structure, do not appear to be latching to the same extent, and the proximal ordered waters differ in their position to the other structures.

Examination of the Neu5Ac bound structure (Fig. 4*C*) explains why *Aa*SiaP prefers Neu5Ac (*K*_D_ = 430 nM) over Neu5Gc (*K*_D_ = 4400 nM). Focusing on the acetyl moiety of Neu5Ac (to which the hydroxyl of Neu5Gc is bound, Fig. 2*C*) shows that it is closely coordinated by the backbone of A66 and F65, the sidechain of N214, and conserved water that is part of the water network (Fig. S6). Neu5Gc binding would either require realignment of the molecule to accommodate the additional hydroxyl, potentially binding in a suboptimal conformation, or disruption of the water network, which is known to be important for binding.

In summary, the conformational changes of SiaP appear to involve: (1) direct and indirect (water-mediated) protein-ligand interactions, (2) the triad of residues connected to the β-strand hinge region, (3) an energetic barrier imposed by the kinked α9 helix, and (4) latching interactions between the two domains.

### MD simulations of the *Aa*SiaP suggest an open state is favored and correlates with hinge straightening

**Figure 5:**
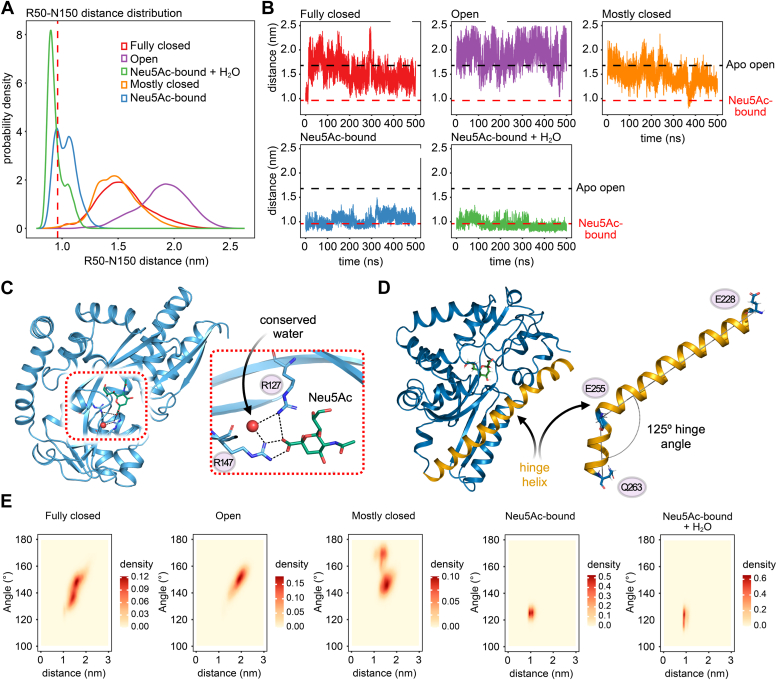
**Molecular dynamic simulation results of *Aa*SiaP.***A*, the distribution of the distance between C_α_ atoms of R50 and N150, calculated from five independent 500 ns MD simulations. Simulations initiated from different conformations of *Aa*SiaP are shown in different colors: fully-closed (*red*), Neu5Ac-bound (*blue*), Neu5Ac-bound with crystallized waters (*green*), open (*purple*), and acetate-bound (*orange*). The *vertical dashed line* indicates the corresponding C_α_ atom distance observed in the crystal structure of Neu5Ac-bound *Aa*SiaP. *B*, dynamic changes in the distance between C_α_ atoms of R50 and N150 across the five simulations. The *horizontal black* and *red dashed lines* represent the distances observed in the apo-open and Neu5Ac-bound crystal structures, respectively. *C*, representation of the highly conserved water molecule within the binding pocket of the *Aa*SiaP Neu5Ac-bound conformation. The inset on the right is a cross-eyed stereo plot of the binding site showing the interaction of the water molecule with the twin-arginine residues (R147 and R127) and the Neu5Ac. *D*, representation of the hinge helix in the crystal structure of Neu5Ac-bound *Aa*SiaP. The inset on the right demonstrates the kink angle of the hinge helix, which is defined by the C_α_ atoms of E228, E255, and Q263. *E*, the probability densities of distances between R50 and N150, alongside the kink angle (as shown in *D*).

Given the possibility that crystal packing may affect the conformation of the protein, we employed full atom MD simulations over 500 ns to determine whether *Aa*SiaP sampled different conformations *in silico*. Our simulations started from five different starting states; three ligand-free states of *Aa*SiaP (open, acetate-bound, and fully-closed with Neu5Ac removed), and two liganded states (the fully-closed Neu5Ac-bound state, and the fully-closed Neu5Ac and conserved water-bound state). The degree of opening was assessed by measuring the distance between the C_α_ atoms of the aforementioned latch residues, R50 and N150 (Fig. 5, *A* and *B*).

The ligand-free simulations starting from the fully closed and acetate-bound states sampled the open state with similar R50-N150 distance distributions (Fig. 5, *A* and *B*, *orange* and *red lines*), whereas the simulation started from the open state sampled a broader distribution of much larger distances (Fig. 5, *A* and *B*, *purple line*). Only the ligand-free state simulation initiated from the acetate-bound state spontaneously sampled closed-state distances, with the simulation initiated from the fully-closed state never returning to closed-state distances, suggesting that while sampling of the closed state by the ligand-free state of *Aa*SiaP in solution is possible, it is uncommon. In contrast, in the Neu5Ac-bound state simulations, the sampled R50-N150 distances are generally close to that of the Neu5Ac-bound crystal structure (Fig. 5, *A* and *B*, *blue* and *green lines*). The second peak in the distance distribution for the simulation without crystallized water molecules in the binding site (blue line) corresponds to the later stages of the simulation where the binding pocket opens slightly, although the Neu5Ac remains bound. A highly conserved water molecule (*i.e.*, observed in other SiaP crystal structures) was found between the twin arginine residues of the binding site (R147 and R127) and the carboxyl oxygen of Neu5Ac (Fig. 5*C*); the R50-N150 distances in the simulation that included this crystallographic water molecule more often correspond to the bound state (Fig. 5, *A* and *B*), suggesting that it may contribute to the affinity of Neu5Ac binding and thus the stabilization of the fully-closed conformation. This is consistent with our crystallographic results above. We note that the water molecule in question is not a part of the Neu5Ac bound water shell (Fig. S5), but rather a structural water molecule. Previous studies demonstrate that both *Hi*SiaP-R147K (PDB id: 2XWI) and *Hi*SiaP-R147A (PDB id: 2XWK) weakly bind Neu5Ac *in vitro*, but in their crystal structures sialic acid is bound as in the wildtype and the protein is in the fully closed state. This indicates that the carboxylate-arginine interactions are not essential for closure, though the simulation data here show that the interaction helps to maintain or stabilize the fully closed state.

To determine whether the opening and closing motion of *Aa*SiaP is related to the bending of the hinge helix α9, the probability densities of the R50-N150 distance and the helix kink angle (Fig. 5*D*) were computed (Fig. 5*E*). The narrow distance distribution observed in the Neu5Ac-bound simulations correlates with the limited flexibility of the hinge helix, which predominantly adopted an angle of ∼125°, even when the binding pocket opened in the simulation without crystallographic water molecules. In contrast, the ∼125° angle of the hinge was only observed at the start of the ligand-free simulation initiated from the fully closed conformation; otherwise, the ligand-free state favored larger hinge angles. Moreover, this transition to wider hinge angles and larger R50-N150 distances at the start of the ligand free-state simulation initiated from the fully-closed state was rapid, and while distances close to that measured for the closed Neu5Ac-bound state are sometimes sampled during the remainder of the simulation, ∼125° hinge angles are not, implying an energy cost associated with maintaining this bound-like hinge angle. The ligand free-state simulations initiated from the fully-closed state had two favored hinge angles of ∼135° and ∼150°, with the latter also favored by the simulation initiated from the open state. In contrast, the simulation initiated from the acetate-bound state favored hinge angles of either ∼145° or ∼170°. Together, these results suggest that while hinge straightening is correlated with the opening of the binding pocket, it is not a straightforward relationship, as the acetate-bound state simulation sometimes samples larger hinge angles than the open state simulation, but the open state simulation generally samples longer R50-N150 distances, representing a more open binding pocket.

### Ligand free-*Aa*SiaP is more open in solution than in the crystal structure

We used small-angle X-ray scattering (SAXS) to investigate the conformational dynamics of *Aa*SiaP in solution, complementing the crystallographic data and molecular dynamic simulations. Although only a low-resolution technique (∼10 Å resolution), SAXS is a label-free approach that is sensitive to small changes in hydrodynamic parameters such as the conformational change expected to occur upon ligand binding [method review here (46)].

We measured scattering data collected in the presence and absence of Neu5Ac. Addition of Neu5Ac resulted in a reduction in the radius of gyration (*R*_g_) and the maximum interatomic distance (*D*_max_): without Neu5Ac the *R*_g_ = 21.31 ± 0.06 Å and the *D*_max_ = 68.1 Å, whereas with 1 mM Neu5Ac the *R*_g_ = 20.43 ± 0.07 Å and the *D*_max_ = 65.0 Å, and with 10 mM Neu5Ac the *R*_g_ = 20.08 ± 0.02 Å and the *D*_max_ = 60.7 Å (Table 3). The significant decrease observed in *R*_g_ and *D*_max_ values from an absence of Neu5Ac in solution to saturating Neu5Ac in solution supports that the protein undergoes a significant detectable conformational change upon ligand binding.

**Table 3:** Summary of structural parameters for *Aa*SiaP from small angle X-ray scattering experiments

SAXS data collection parameters	
Instrument	Australian Synchrotron SAXS/WAXS beamline
Detector	PILATUS 1M (dectris)
Wavelength (Å)	1.0332
Maximum flux at sample	8 × 10^12^ photons per second at 12 keV
Camera length (mm)	1600
q-range (Å^−1^)	0.006–0.5
Exposure time	Continuous 1 s frame measurements
Sample configuration	SEC-SAXS with co-flow
Sample temperature (°C)	12


SAXS data analysis	Apo	Neu5Ac (0.1 mM)	Neu5Ac (10 mM)
Guinier analysis			
*I*_*o*_ (cm^−1^)	0.029 ± 4.5 × 10^−5^	0.029 ± 5.7 × 10^−5^	0.053 ± 3.1 × 10^−5^
*R*_*g*_ (Å)	21.31 ± 0.06	20.43 ± 0.07	20.08 ± 0.02
*P*(*r*) analysis			
*I*_*o*_ (cm^−1^)	0.03	0.03	0.05
*R*_*g*_ (Å)	21.30	20.33	19.99
*D*_max_ (Å)	68.1	65.0	60.7
Porod volume (Å^−3^)	47,313.3	51,858.8	51,187.3
CRYSOL analysis			
Open (X^2^ value)	0.708/0.949	0.497/0.513	7.317/11.067
Acetate-bound (X^2^ value)	2.203/2.291	0.759/0.775	0.375/0.482
Closed (X^2^ value)	2.583	0.873	0.366
SAXS-MoW2^a^			
Estimated MW (kDa)	28.5	23.2	28.1


Crysol prediction	Chain C (open)	Chain D (open)	Chain A (closed)	Chain B (closed)	Bound *Aa*SiaP
*R*_*g*_ (Å)	20.48	20.22	20.07	19.99	19.7
*D*_max_ (Å)	72.17	70.93	71.13	68.14	66.81

a http://saxs.ifsc.usp.br/.

**Figure 6:**
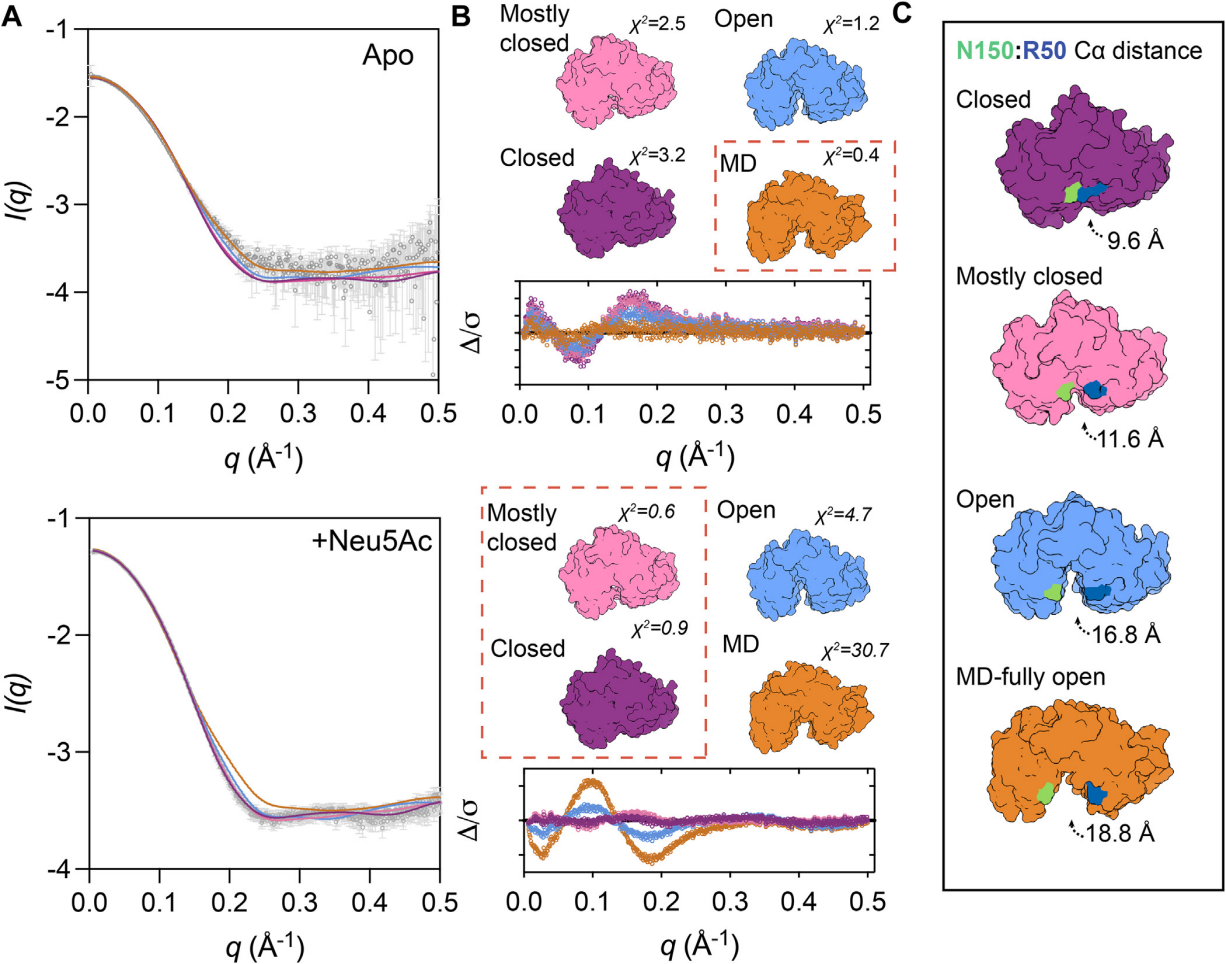
**Small angle X-ray scattering analysis of *Aa*SiaP.***A*, *Aa*SiaP scattering data (*grey*), fitted with the theoretical scattering curves from various models using *Crysol* (70). Data were collected in the absence of Neu5Ac (*top*, ligand-free); data were collected in the presence of 10 mM Neu5Ac (*bottom*). *B*, error-weighted residual plots of the fits, alongside the structural models used to generate the fits. The best fits to the data (lowest χ^2^) are indicated by the *red dashed box*. *C*, the large extent of the conformational change between the closed structure and a fully open MD model. The distance displayed is the distance between the C_α_-atoms of R50 and N150, a residue pair proposed to latch together (45).

The experimental scattering data were fit with theoretical scattering curves calculated from the X-ray crystal structures (Fig. 6, *A* and *B*). As expected, the data collected with Neu5Ac (10 mM) were best fit by the closed structure, whilst data without Neu5Ac were initially best fit by the open structure. Fitting fully open MD-based models (presented in the previous section) to our SAXS data gave an even better fit than the open structure (Fig. 6*B*). Further analysis of the fully open MD model corroborates our previous hypothesis that R50 and N150 form part of the latch, which moves significantly further apart from closed, to acetate-bound, to open, to fully open structures (Fig. 6*C*). Examination of the crystal packing for the unbound-*Aa*SiaP structure (Fig. S7) suggests that the formation of the wider conformation is constrained by crystal contacts and explains why in this crystal form we do not observe this conformation, but which is evidenced by the MD simulations and SAXS data. Overall, our SAXS and MD analyses support that the ensemble average of *Aa*SiaP conformations sampled in solution is more open than the open conformation determined in the crystal structure, although the MD analysis suggests that the open conformation can (rarely) sample the closed conformation.

## Discussion

### New structures suggest acetate molecules can induce only partial closure of *Aa*SiaP

We report two crystal structures of *Aa*SiaP that show three conformations: (1) a fully closed conformer with Neu5Ac bound, (2) an open conformer that is similar to previously reported ligand-free structures, (3) a unique ligand-free conformer that is acetate-bound. The ligand free-*Aa*SiaP crystal structure contains two conformations of *Aa*SiaP. Ligand free-*Aa*SiaP crystallized in acetate-containing conditions (200 mM) and the electron density in the binding site is more consistent with acetate than a water molecule or Neu5Ac. The crystal contacts/interfaces in the ligand free-*Aa*SiaP structure are largely the same and therefore unlikely to explain the observed difference in conformation.

**Figure 7:**
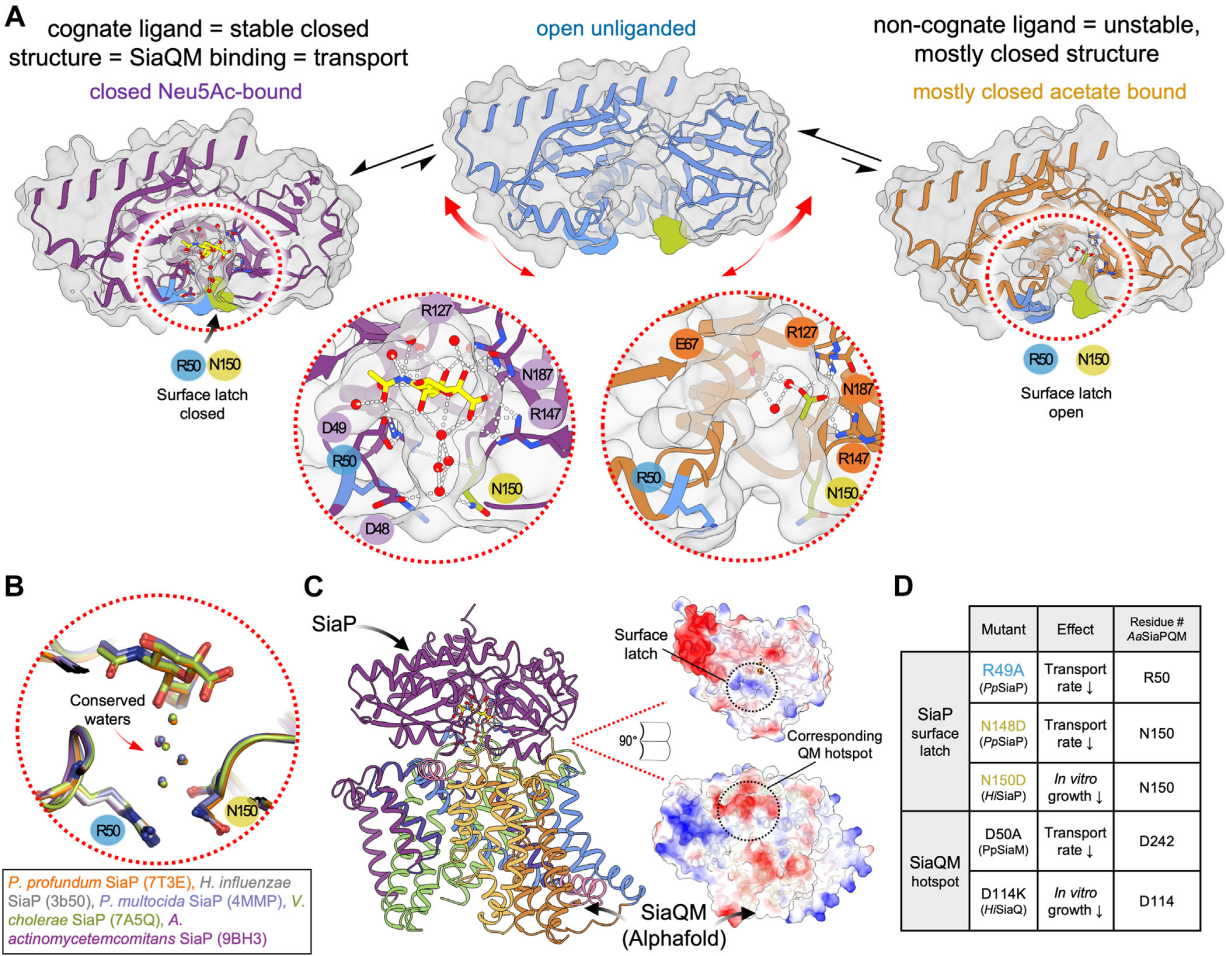
**Full closure of SiaP involves latching and a water network that extends to the interaction surface**. *A*, the open unliganded form of *Aa*SiaP can sample a range of conformations including (largely) a more open structure that is not found in the crystal, and (rarely) the closed conformation. Binding Neu5Ac and a water network stabilizes the closed conformation that can bind to the SiaQM membrane components. In contrast, binding a non-cognate ligand leads to an acetate-bound conformation, without a stabilized water network and presumably cannot bind the SiaQM membrane components. *B*, structural overlay of SiaP homologs showing the similar positioning of the latch residues and water network. *C*, alphafold2 modeling of *Aa*SiaP-SiaQM, with an open-book representation of the interacting surfaces. *D*, table showing the functional effects of mutating residues at either the surface latch region or the corresponding region on SiaQM that interacts with the latch residues. These data are drawn from previous studies (17, 18).

We propose that the binding of acetate can stabilize only partial closure, which is consistent with the induced fit model of substrate binding, in that the non-cognate ligand is unable to induce the tight binding (*i.e.*, closed) conformation (Fig. 7*A*). The specificity and sensitivity of SiaP for Neu5Ac have been well-established in the literature, with even small modifications to the Neu5Ac structure having significant effects on binding affinity (27). Closure around a non-cognate ligand is consistent with models proposed for other substrate-binding proteins, where their binding induces a partially closed conformation that is “conformationally mismatched” to the membrane domains, so does not facilitate transport (47). Recently, Peter *et al.* (2021) reported a structure of triggering (37). The authors sensibly propose that full closure is triggered by the physical bridging of the two domains by the ligand. Our “mostly closed” *Aa*SiaP structure with acetate bound suggests that the carboxylate interaction may be sufficient to induce closure, but not a full “latching” between domains.

Considering now the proposed mechanism of TRAP transporter import, we suggest that failure to induce full closure by non-cognate ligands is a method of discrimination for SiaPQM systems, in that only the cognate ligand induces a fully closed conformation that can productively interact with the membrane domains to facilitate transport (as illustrated in Fig. 7*A*), which has been proposed in the ABC family transporters (47).

### Ligand free-*Aa*SiaP displays conformational heterogeneity and closure in solution

Our MD simulations demonstrate that the ligand-free state strongly favors an open state—in terms of both the R50-N150 distance and the α9 helix hinge angle—regardless of the structure from which the simulation is initiated. These simulations also suggest that the R50-N150 distances are often larger than in the ligand-free open-state crystal structure and that near-closed conformations can be sampled, although rarely. The small angle X-ray scattering data also suggest that in solution at biologically relevant temperatures, ligand-free *Aa*SiaP exists predominantly in an open conformation that is likely more open than in the open-state crystal structure. This is consistent with findings from PELDOR spectroscopy experiments on frozen *Vc*SiaP (36) and atomistic MD simulations based on this PELDOR data, which suggest that *Vc*SiaP in solution may also be more open than in the open-state crystal structure (48). More recently, rare closure events have been reported for a ligand-free disulfide-engineered *Hi*SiaP construct, where residues within the latch region of SiaP are substituted (S15C/A194C) (49). In this experiment, disulfide formation between these residues only occurs when SiaP closes and positions the residues in close contact with the required geometry, which was detected as a distinct band shift by non-reducing SDS-PAGE in the absence of oxidizing agents. A time-course experiment based on this band shift, quantifying the relative proportions of non-disulfide and disulfide-linked proteins suggests that the ligand-free *HiS*iaP protein underwent an intrinsic closing motion approximately every 23 s. This is equivalent to less than 0.01% of all molecules closing within one millisecond, and since the ligand-free SiaP quickly reopens in solution, only a small fraction of closed ligand-free SiaP would be present at one time. This is generally supported by our MD simulations, which only show partial closure over the 500 ns simulation time.

In contrast, the closed conformation is strongly favored when Neu5Ac is bound, but the conserved water molecule between R127 and R147 is required to fully stabilize this state. This data fits nicely with recent work that demonstrates the importance of water networks for Neu5Ac binding (25). Our crystal structure of the Neu5Ac bound shows a highly ordered network of waters in the ligand binding site that coordinates both the ligand and the protein. Many of these waters are highly conserved in other Neu5Ac-bound SiaP structures.

### Implications for the formation of the SiaP-SiaQM complex and substrate release

A prominent model proposed by Mulligan *et al.* suggests that only the closed-bound SiaP conformation productively binds the SiaQM membrane domains, which in turn modulates SiaP into an open conformation, prompting the transfer of the substrate (24). More recently, Peter *et al.* (2021) reported SiaP closure and binding to peptides based on the predicted periplasmic loops of the SiaQ subunit. These findings are consistent with the “scoop-loop model” of some ABC transporters, where the periplasmic loops of the membrane domains help dislodge the bound substrate and/or prevent the released substrate from reassociating with the substrate-binding protein instead of the membrane domains (50). However, modeling of the SiaP-SiaQM interaction does not point to an obvious scoop-loop, even considering large elevator-type movements of the transport domain (17). We speculate that it is more likely that substrate release is governed by SiaP-SiaQM surface interactions that disrupt the Neu5Ac binding site allosterically.

Evidence that the SiaP and SiaQM domains are conformationally coupled has come from Peter *et al.* (49), who assessed interactions for *Hi*SiaQM disulfide engineered constructs locked in the inward-facing state (IFS) and outwards-facing state (OFS) mixed with both wildtype and the disulfide engineered *Hi*SiaP constructs (described above). Their findings largely support a transport model where the closed SiaP preferentially interacts with the SiaQM IFS and the open SiaP preferentially interacts with the SiaQM OFS. Exactly how SiaP interacts with the OFS needs investigation, and in particular, whether or not SiaP is fully open during this interaction. It may be possible that SiaP adopts an intermediate open state while bound to the OFS, considering that the closed SiaP could still occasionally interact with the OFS (49). How the fully open state observed here fits in with the mechanism is unclear, though we speculate that this state should have the lowest affinity for SiaQM and that this promotes dissociation from the transporter. Further structural and functional studies are needed to better assess these interactions.

Our data suggests conserved water networks and residues involved in coordinating Neu5Ac are required to give the fully closed P domain (Fig. 7, *A* and *B*), which presumably has the highest affinity for the inward-facing SiaQM domains (Fig. 7*C*). We report partial closure with the co-ordination of the non-cognate ligand acetate to key residues within the Neu5Ac binding site, but this partial closure was insufficient to induce latch closure. Residues involved in this latch are critical to interactions with the SiaQM domains and for transport activity (17) (Fig. 7*D*). Overall, our data support that the substrate-binding induced latch formation may be a key requisite for high-affinity SiaP docking and subsequent substrate handover to SiaQM.

To conclude, we report an acetate-bound-non-cognate bound conformation of AaSiaP similar to structures previously reported for ABC substrate-binding proteins (32, 47, 51), but which has not to our knowledge been observed for TRAP substrate-binding proteins. We propose that interactions between residues at the resulting domain interface stabilize the acetate-bound-unbound conformation in equilibrium and that ligand-binding induces small but important residue movements that mediate productive interactions with the membrane domains. These results are inconsistent with a simple two-state induced-fit model of ligand binding; thus, we instead propose a special case of induced fit that involves an intrinsic conformational change in the absence of a ligand. Our findings contribute to a deeper understanding of the conformational dynamics of SiaP proteins and other TRAP substrate-binding proteins, which may aid in the design of novel inhibitors for use as antimicrobial drugs.

## Experimental procedures

### Multiple sequence alignment

The position-specific iterated protein (PSI-)BLAST was performed using the *A. actinomycetemcomitans* SiaP protein sequence (NCBI accession #WP_005538762.1) as the query to identify homologous substrate-binding proteins. Twelve of the highest-scoring sequence identity sequences from a diverse range of species, including four proteins with available PDB structures (Fig. 1), were chosen for sequence alignment and structural comparison (Fig. S1): NCBI accession no. WP_109077085.1, 2CEY_A, WP_077422743.1, WP_100297742.1, WP_132021368.1, WP_039082755.1, 4MMP_A, WP_075321769.1, WP_048716221.1, WP_103853354.1, 4MNP_A, 4MAG_A. Multiple sequence alignment was performed using Clustal Omega (https://www.ebi.ac.uk/Tools/msa/clustalo/) (52) and the figure was produced in ESPript 3.0 (http://espript.ibcp.fr/ESPript/cgi-bin/ESPript.cgi) (53).

### Transformation and protein production

The *A. actinomycetemcomitans* strain 624 *siaP* gene (NCBI accession no. NZ_CP012959, Region: 361430–362416) was synthesized by Genscript and ligated into expression vector pET22B(+). In this construct, the native periplasmic signal peptide sequence, as predicted by the SignalP-5.0 server (54), was replaced with *pelB* periplasmic signal peptide sequence from *Erwinia carotovora*. A residual two-residue (methionine and aspartate) cloning fragment was retained at the N-terminus of the mature chain following removal of the 22-residue *pelB* leader sequence. Chemically competent *E. coli* BL21 (DE3) cells were then transformed with recombinant pET22B(+) containing the *A. actinomycetemcomitans siaP* sequence and allowed to recover in super optimal broth with catabolite repression at 37 °C for 1 h. Recombinant cells were then plated on Luria-Bertani (LB) agar medium supplemented with ampicillin (100 μg/ml) and incubated overnight at 37 °C. After being confirmed, the recombinant strain was stored at −80 °C.

Starter cultures of the recombinant strain were prepared from a single colony, which was inoculated into LB medium supplemented with ampicillin (100 μg/ml) and incubated for 4 h at 37 °C and 180 rpm. Following incubation, the culture was centrifuged at 4230*g* for 10 min and the resulting cell pellet was washed with 1 × M9 salts, then inoculated into M9 medium supplemented with ampicillin (100 μg/ml) and incubated overnight at 37 °C and 180 rpm. For protein production assays, a starter culture was used to inoculate a 4 L volume of M9 minimal medium supplemented with ampicillin (100 μg/ml), which was then incubated at 26 °C for ∼3 h to an optical density at 600 nm of 0.3. To induce protein production, isopropyl β-d-1-thiogalactopyranoside was added to a final concentration of 1 mM, and the culture was incubated at 16 °C overnight. Following incubation, the cells were collected by centrifugation at 5600*g* for 10 min and resuspended in periplasmic extraction buffer (50 mM Tris-HCl, 500 mM sucrose, 1 mg/ml lysozyme, pH 8.0) at a concentration of 5 ml/g cell weight. DNase (1 μg/ml) was added to the cell suspension and the mixture was incubated for 1 h at 4 °C. Following incubation, 10 ml of ice-cold water was added, and the lysate was further incubated for 30 min.

### Protein purification

All purification steps were performed at 4 °C, and samples were maintained on ice throughout. Anion-exchange chromatography (AEX) was performed using a Q Sepharose Fast Flow Column (HiPrep Q FF 16/10; GE Healthcare) pre-washed with three column volumes of AEX Buffer B (20 mM Tris-HCl, 1 M NaCl, pH 8.0) to elute bound proteins. The column was then equilibrated with three column volumes of AEX Buffer A (20 mM Tris, pH 8.0). The collected periplasmic fraction was loaded onto the column and bound recombinant *Aa*SiaP was eluted using an increasing gradient of AEX Buffer B over 10 column volumes.

Hydrophobic interaction chromatography (HIC) was then conducted using a Phenyl Sepharose 6 Fast Flow Column (HiPrep Phenyl FF (High Sub) 16/10, GE Healthcare) pre-washed with three column volumes of HIC Buffer B-SEC Buffer (20 mM Tris-HCl, 150 mM NaCl, pH 8.0) and equilibrated with three column volumes of HIC Buffer A (20 mM Tris, 1 M (NH_4_)_2_SO_4_). Ammonium sulfate (final concentration, 1 M) was added to the pooled *Aa*SiaP-containing fractions, which were then loaded onto the Phenyl Sepharose column. Bound *Aa*SiaP was again eluted using an increasing gradient of HIC Buffer B over 10 column volumes.

Size-exclusion chromatography (SEC) was employed as the final purification step using a Superdex 200 Column (Superdex 200 Increase 10/300 Gl, GE Healthcare) pre-equilibrated with three-column volumes of SEC buffer (Tris-NaCl). The pooled fraction from the HIC step was concentrated to ∼40 mg/ml (Millipore) and then loaded onto the column. *Aa*SiaP-containing fractions were pooled, and purification was confirmed by sodium dodecyl sulphate polyacrylamide gel electrophoresis analysis. Protein concentration was estimated by measuring absorbance at 280 nm and using an extinction coefficient of 23,840 M^−1^·cm^−1^. Purified protein that was not used immediately was flash-cooled in liquid nitrogen and stored at −80 °C. The typical final yield of *Aa*SiaP was ∼30 mg/L of culture.

### Differential scanning fluorimetry

Purified *Aa*SiaP in SEC buffer was concentrated using Vivaspin centrifugal concentrators (10,000 Da molecular weight cut-off; Sartorius) at 8000*g* then subjected to two rounds of buffer exchange using equimolar Tris-KCl buffer and a 5 mL desalting column (HiTrap Desalting; GE Healthcare) pre-equilibrated with four column volumes of Tris-KCl. Differential scanning fluorimetry was conducted as described (55), with some modifications, using a QuantStudio 3 Applied Biosystems real-time polymerase chain reaction (PCR) system with differential scanning fluorimetry capability (Thermo Fisher Scientific). Assays were repeated in triplicate in a 96 well 0.2 mL PCR-compatible plate. The 20 μl reaction volumes contained 16 μl of Tris-NaCl/KCl buffer (±Neu5Ac at various concentrations), 1 μM *Aa*SiaP (2 μl), and 50 × SYPRO orange dye (2 μl). Neu5Ac was dissolved in small aliquots of buffer in triplicate to achieve concentrations ranging from 0.00025 to 1 mM, corresponding to the expected *K*_D_ range. Samples were gradually cooled from an initial temperature of 29 °C to 20 °C and held for 1 min. Samples were then heated incrementally to 95 °C at a rate of 0.015 °C/s (∼84 min), and fluorescence intensity was recorded over time.

### Fluorescent labeling and microscale thermophoresis

For subsequent microscale thermophoresis (MST) binding experiments, *Aa*SiaP was labeled at pH 8.0 (the lower end of the recommended pH range for labeling), which directed fluorescein 5-isothiocyanate (FITC) towards the lower pKa α-amino at the N-terminus. This was done to minimize the amount of structural and conformational perturbation, as the protein contains 29 lysine residues that would be more reactive at higher pH. *Aa*SiaP (30 μM) in storage buffer (Tris-HCl 50 mM, NaCl 150 mM, pH 8) was buffer exchanged into PBS, pH 7.4, incubated with FITC (1:40 M ratio *Aa*SiaP:FITC in DMSO) for two hours at room temperature, then spin concentrated iteratively (3 kDa molecular weight cut-off) to remove free dye before the separation of the *Aa*SiaP-FITC conjugate by SEC, as detected by absorbance at 280 nm and 490 nm. Fluorophore/protein (F/P) molar ratio was determined from absorption at these wavelengths and calculated by the equation: M F/P = (A_495_ × C)/(A_280_ – [(0.35 × A_495_)], where C is a constant for the protein: C = (MW × E0.1%)/(389 × 195) = 34,000 g/mol × 0.693/389 × 195 = 0.3106), which gave a F/P ratio of 0.42. MST binding experiments were performed on a Monolith NT.115 (NanoTemper Technologies) with Monolith NT.115 Capillaries. Binding checks were performed with various concentrations of *Aa*SiaP with both Neu5Ac and Neu5Gc, which suggested that 10 mM Neu5Ac (100 fold higher than the highest concentration in the binding assay) suggested ligand autofluorescence at this excess concentration; low fluorescent counts were also observed for some replicates at lower concentrations, which we attribute to the low labelling ratio, so a higher concentration of 75 nM *Aa*SiaP was selected for binding affinity experiments. Binding affinity experiments were performed in duplicate (Neu5Gc) or triplicate (Neu5Ac) at 16 different concentrations of ligand, ranging from 100 μM to 0.00305 μM, and analyzed with MO Affinity Analysis V2.3 software.

### Analytical ultracentrifugation

Sedimentation velocity analytical ultracentrifugation was performed using a Beckman Coulter ProteomeLab XL-1 Protein Characterisation System, with a Beckman Coulter AN50Ti rotor with sapphire and quartz windowed cells. Data were collected at 20 °C with absorbance optics (280 nm) and run at 42,000 rpm. Recombinant *Aa*SiaP at 0.42 mg/ml (12 μM, concentration measured by absorbance at 280 nm) was analyzed in Tris-NaCl buffer in the absence of Neu5Ac, and with two concentrations of Neu5Ac (0.1 mM and 0.5 mM).

Data were analyzed using UltraScan 4.0, release 2578 (56). Sedimentation data were evaluated by the two-dimensional spectrum analysis (2DSA) (57, 58), with simultaneous removal of time- and radially invariant noise contributions and fitting of boundary conditions. In addition, 50 Monte-Carlo iterations were performed on the 2DSA data set and 2DSA-Monte-Carlo solutions were subjected to parsimonious regularization by genetic algorithm analysis (59). All fitting procedures were completed using the UltraScan LIMS cluster. Each genetic algorithm model was visually assessed to ensure a good fit and a low RMSD using the FE Model Viewer utility in UltraScan (Fig. S4). Predicted values for hydrodynamic parameters were estimated in *Hullrad* (60) for *Aa*SiaP crystal structures.

### Crystallization and structure determination

Crystallization trials were set up using the Clear Strategy, ShotGun (SG1), and PACT premier screens in 96 well plates. Drops consisting of 400 nl of mother-liquor and protein solution (*Aa*SiaP at 20 mg/ml along with and without 0.75 mM Neu5Ac in SEC buffer) were mixed using the Mosquito Protein Crystallization System and the sitting-drop vapor-diffusion method and incubated at 20 °C. Apo *Aa*SiaP crystals that grew in SG1 conditions C11 (0.2 M sodium acetate trihydrate, 0.1 M sodium cacodylate, pH 6.5, 30% (w/v) PEG 8000) and F2 (0.2 M sodium acetate trihydrate, 0.1 M sodium HEPES, pH 7.5, 25% (w/v) PEG 3350) were mounted in loops and preserved in 15% (v/v) cryoprotectant (50% (v/v) ethylene glycol, 50% (v/v) glycerol). Neu5Ac (0.75 mM) bound *Aa*SiaP crystals that grew in SG1 condition H2 (30% w/v PEG 4000) were not cryo-protected before being flash-cooled in liquid nitrogen prior to data collection.

The diffraction data for recombinant *Aa*SiaP were collected at the Australian Synchrotron (Melbourne, VIC) on the MX2 beamline using an X-ray wavelength of 0.91840 Å equipped with an EIGER 16 M detector. The apo crystals from SG1 condition C11 diffracted to a maximum resolution of 2.58 Å and belonged to space group *C*2. In contrast, crystals grown in SG1 F2 diffracted to a maximum resolution of 3 Å and also belonged to space group *C*2. Crystals from SG1 H2 diffracted to a maximum resolution of 1.90 Å and belong to space group *P*2_1_. Diffraction data were scaled and processed using *XDS* (60) and *Aimless* (*CCP4i2 suite*) (61). Resolution cut-offs were determined following the criteria that the CC_1/2_ of each dataset is above 0.35 and where the *I/σI* was equal to or greater than 1.0 (62). Data collection statistics are reported in Table 2.

Structures were determined by molecular replacement using *Phaser* (CCP4i2 suite) (63). Apo *Aa*SiaP was solved with four molecules per asymmetric unit, two in an open conformation and two in a near-closed conformation, using two different search models: an apo-structure of *H. influenzae* SiaP (PDB ID: 2CEY) edited using *Sculptor* (CCP4i2 suite), and the structure of Neu5Ac-bound *Aa*SiaP. The structure of Neu5Ac-bound *Aa*SiaP was solved by molecular replacement using a liganded structure of *H. influenzae* SiaP (PDB ID: 2WYK) with two protein molecules in the asymmetric unit. For both structures, initial manual model rebuilding was done in *Coot* (64) and refined using *Refmac* (CCP4i2 suite) (65, 66). The resulting data was then exported to *Phenix* (67, 68) for refinement, with an initial round of refinement performed by simulated annealing (cartesian) to reduce model bias. Refinement with translation-libration-screw (TLS groups were determined automatically using *Phenix*). Water molecules were identified and added in the later stages of refinement. The final model for apo-*AaSiaP* included two monomers in the open conformation (chains C and D) and two monomers in a near-closed conformation (chains A and B). Weak density was observed for cloning fragments at residues 1 and 2 at the N-terminus. Refinement statistics are presented in Table 2.

### Small-angle X-ray scattering

Purified *Aa*SiaP in Tris-NaCl (SEC buffer) was spin-concentrated at 8000*g* using Vivaspin centrifugal concentrators (10,000 Da molecular weight cut-off; Sartorius) before being subjected to buffer exchange into SAXS buffer (20 mM Tris, 50 mM NaCl, pH 8) using a 5 ml HiTrap desalting column pre-equilibrated with three column volumes of SAXS buffer.

Small-angle X-ray scattering (SAXS) data were collected on the SAXS/WAXS beamline equipped with a Pilatus 1 M detector (170 × 170 mm, effective pixel size, 172 × 172 μm) at the Australian Synchrotron (69). A sample detector distance of 1600 mm was used, providing a *q* range of 0.05 to 0.5 Å^−1^. Here 70 μl of purified *Aa*SiaP protein at 10 mg/ml was injected onto an inline Superdex S200 Increase 5/150 Gl (GE Healthcare) SEC column, equilibrated with 20 mM Tris–HCl pH 8.0, 150 mM NaCl, supplemented with 0.1% (*w*/*v*) sodium azide, using a flow rate of 0.45 ml·min^−1^. For data collected in the presence of Neu5Ac, the same buffer was used with Neu5Ac added to a final concentration of 0.1 or 10 mM. Scattering data were collected in one second exposures (λ = 1.0332 Å) over a total of 400 frames, using a 1.5 mm glass capillary, at 8 °C. 2D intensity plots were radially averaged, normalized to sample transmission, and background subtracted using the *Scatterbrain* software package (Australian Synchrotron), and then analyzed using *Chromixs* in the ATSAS suite (70). The theoretical scatter, *R*_*g*_ and *D*_max_ values for the open, near-closed, and closed-liganded conformations were calculated in *Crysol* (71) and compared to the experimental data.

Prior to *ab initio* shape determination, the ambiguity score for each condition was calculated in *Ambimeter* (72); in all cases, the score was less than 1.5 (1.146, 0.9542, and 0.7782 for 0, 0.1, and 10 mM Neu5Ac respectively) indicating that unique non-ambiguous shapes could be determined from the experimental scatter. Ab initio bead models were then created in ten independent *Gasbor* (73) runs (accessed *via* https://www.embl-hamburg.de/biosaxs/atsas-online/) and the representative models, as determined by *Damaver* and *Damfilt*, were superimposed onto the *Aa*SiaP crystal structures using *Subcomb* (74) *via* the *SASpy* plugin (75) for open source *PyMOL*. The parameters for data collection and processing are summarized in Table 3.

### Molecular dynamics simulations

All simulations were performed using the GROMACS 2021.5 software package (76) and the CHARMM36m force field (77); the integration time-step was 2 fs, and periodic boundary conditions were applied. The parameters for Neu5Ac were obtained from the CHARMM36m ligand database. Short-range electrostatic and van der Waals interactions were cut-off at 1.2 nm; the particle mesh Ewald method (78) was used to treat long-range electrostatic interactions and the van der Waals interactions were force-switched from 1.0 to 1.2 nm. Non-bonded neighbor lists were updated using the Verlet cutoff scheme (79). The LINCS algorithm (80) was used to constrain the hydrogen bond lengths to their equilibrium value. The temperature was maintained at 298 K and the pressure at 1 bar using the velocity-rescale thermostat (81) and the Berendsen barostat (82), respectively, aside from during the reduction simulation, which used the Parrinello-Rahman barostat (83). Each production simulation lasted 500 ns, with coordinates saved every 5 ps.

#### Simulation preparation

In all systems, the energy was first minimized using the steepest descent algorithm until the maximum force changed by less than 1000 kJ·mol^−1^·nm^−1^, and then the system was solvated using the TIP3P water model (84) (Table S4). Na^+^ and Cl^−^ ions were then added to neutralize the system (Table S4), followed by again minimizing the systems energy. Initial velocities of each system were randomly generated from a Maxwell-Boltzmann distribution at 50 K in the NVT ensemble, followed by a 10 ps equilibration and then heating smoothly from 50 K to 298 K over 210 ps, and a second brief equilibration at 298 K for 40 ps. Each system was then equilibrated for 250 ps with the temperature and pressure maintained at 298 K and 1 bar.

#### Analysis

Simulation trajectories were visualized using Visual Molecular Dynamics (85) and images made using Pymol (The PyMOL Molecular Graphics System, Version 3.0 Schrödinger, LLC). Distances and angles were calculated using the GROMACS analysis tools *gmx mindist* and *gmx gangle*, respectively, and the probability densities were calculated and plotted using *R 4.3.2* (86). The minimum distance was between the Cα atom of residues R50 and N150, and the helix kink angle was defined as the angle between the vectors connecting the Cα atoms of residues Q263-E225 and E225-E228.

## Data availability

All data will be made available on request to the corresponding author. X-ray crystal structures and data are accessible through the Protein Data Bank [PDB IDs: 9BH3 (Ligand free-*Aa*SiaP) and 9BHF (Neu5Ac-bound *Aa*SiaP)].

## Supporting information

This article contains supporting information (2, 8, 17, 18, 25, 26, 27, 28, 36, 37, 45).

## Conflict of interest

The authors declare that they have no conflicts of interest with the contents of this article.
