# Supplementary material for: On the function of TRAP substrate-binding proteins: Conformational variation of the sialic acid binding protein SiaP
Source: J Biol Chem. 2024 Sep 30;300(11):107851. doi: 10.1016/j.jbc.2024.107851 (PMC11550005; doi:10.1016/j.jbc.2024.107851)
Supplement: Supporting Information [file mmc1.docx]

Supporting Information, Tables, and Figures for:

# *On the function of TRAP substrate-binding proteins: conformational variation in the sialic acid binding protein SiaP*

# *Te-Rina J. King-Hudson, James S. Davies, Senwei Quan, Michael J. Currie, Zachary D. Tillett, Jack Copping, Santosh Panjikar, Rosmarie Friemann, Jane R. Allison, Rachel A. North, Renwick C.J. Dobson*

# Supplementary Tables

**Supplementary Table 1 |** Summary of binding affinity data published for SiaP orthologues (as of April 2024).

| reference | org | ligand | *K*_D_ (µM) | mutation | technique |
| --- | --- | --- | --- | --- | --- |
| (25) | Hi | Neu5Ac | 0.030 ± 0.012 | - | ITC |
|  |  | Neu5Gc | 0.138 ± 0.013 | - |  |
|  |  | KDN | 8.3 ± 1.5 | - |  |
|  |  | Neu5Ac | 42 ± 2.8 | A11N |  |
|  |  | Neu5Gc | 31 ± 0.4 | A11N |  |
|  |  | KDN | 497 | A11N |  |
| (26) | Hi | Neu5Ac | 0.11 ± 0.02 | - | ITC |
|  | Hi | Neu5Ac | 0.14 ± 0.04 | His tag | Fluorescence spectroscopy |
|  |  | Neu5Ac | No binding | R147A |  |
|  |  | Neu5Ac | No binding | R147K |  |
|  |  | Neu5Ac | 1.21 ± 0.03 | F170W |  |
|  |  | Neu5Ac | 38,700 ± 8,300 | F170W / R147K |  |
| (27) | Hi | Neu5Ac | 0.058 ± 0.005 | - | Steady-state fluorescence spectroscopy |
|  |  | Neu5Ac (no Na) | 0.138 ± 0.006 |  |  |
|  |  | Sialylamide | 243 ± 28 | - |  |
|  |  | dNeu5Ac | 34 ± 2.5 | - |  |
| (2) | Hi | Neu5Ac | 0.12 ± 0.01 | - | Steady-state fluorescence spectroscopy |
|  |  | Neu5Gc | 0.29 ± 0.04 | - |  |
|  |  | α-2,3-sialyllactose | 18 ± 0.3 | - |  |
|  |  | Neu5Ac2en | 20 ± 3.8 | - |  |
|  |  | KDN | 42 ± 3.0 | - |  |
|  |  | Neu5Ac (NH_4_Ac pH 5, no Na) | 0.12 ± 0.02 | - |  |
| (28) | Hi | Neu5Ac | 0.0166 | - | MicroCal ITC |
|  |  | Neu5Ac | 19.8 | E67A |  |
|  |  | Neu5Ac | 1.78 | E186Q |  |
|  |  | Neu5Ac | 1.41 | N187Q |  |
|  |  | Neu5Ac | 0.641 | T64K |  |
|  |  | Neu5Ac | 0.662 | H209A |  |
|  |  | Neu5Ac | No binding | T64R |  |
|  |  | Neu5Ac | No binding | R127K |  |
| (45) | Hi | Neu5Ac | 0.11 ± 0.02 | His tag | Fluorescence spectroscopy |
|  |  | Neu5Ac | 980 ± 190 | R127K, His tag |  |
|  |  | Neu5Ac | No binding | R127E, His tag |  |
|  |  | Neu5Ac | No binding | R127A, His tag |  |
|  |  | Neu5Ac | No binding | R147K, His tag |  |
|  |  | Neu5Ac | No binding | R147A, His tag |  |
|  |  | Neu5Ac | No binding | R147E, His tag |  |
|  |  | Neu5Ac | No binding | N187A, His tag |  |
|  |  | Neu5Ac | No binding | N187D, His tag |  |
|  |  | Neu5Ac | 1500 ± 300 | F170W; R127K, His tag | Fluorescence spectroscopy |
|  |  | Neu5Ac | No binding | F170W;R127E, His tag |  |
|  |  | Neu5Ac | No binding | F170W;R127A, His tag |  |
|  |  | Neu5Ac | No binding | F170W;R147K, His tag |  |
|  |  | Neu5Ac | No binding | F170W;R147A, his tag |  |
|  |  | Neu5Ac | No binding | F170W;R147E, His tag |  |
|  |  | Neu5Ac | 244 ± 8 | F170W;N187A, His tag |  |
|  |  | Neu5Ac | No binding | F170W;N187D, His tag |  |
|  |  | Neu5Ac | 15 ± 7 | S15D;A195D, His tag | ITC |
|  |  | Neu5Ac | 76 ± 4 | S15D;A195K, His tag |  |
|  |  | Neu5Ac | 9.3 ± 1.9 | S15K;A195D, His tag |  |
|  |  | Neu5Ac | 38 ± 9 | S15K;A195K, His tag |  |
|  |  | Neu5Ac | 580 ± 190 | Q72E;A151K, His tag |  |
|  |  | Neu5Ac | 3.4 ± 1.3 | Q72E;A152K, His tag |  |
|  |  | Neu5Ac | 0.10 ± 0.02 | N150D*, His tag | * abolished transport, *in vitro* uptake assay |
| (8) | Vc | Neu5Ac | 0.028 ± 0.001 | - | ITC |
| (18) | Vc | Neu5Ac | 0.300 ± 0.100 | Q54C-R1A / L173C-R1A | Solution-based FRET (titration) |
|  |  | Neu5Ac | 0.203 ± 0.017 | Q54C-R1A / L173C-R1A | ITC |
| (28) | Vc | Neu5Ac | 0.306 | - |  |
|  |  | Neu5Gc | 1.090 | - |  |
| (36) | Vc | Neu5Ac | 0.8 ± 0.4 | Q54C-R1 / L173C-R1 | PELDOR Spectroscopy |
| (28) | Fn | Neu5Ac | 0.0455 | - | MicroCal ITC |
|  |  | Neu5Gc | 0.0457 | - |  |
|  | Pm | Neu5Ac | 0.0197 | - |  |
|  |  | Neu5Gc | 0.0307 | - |  |

**Supplementary Table 2 |** Summary of published SiaP structures on RCSB PDB (as of April 2024). Of the 22 structures deposited in the PDB, 15 are variation on *Hi*SiaP (mutants, different ligands) and four are *Vc*SiaP.

| reference | PDB | organism | resolution (Å) | space group | substitution | ligands |
| --- | --- | --- | --- | --- | --- | --- |
| (17) | 7T3E | Pp | 1.04 | P 2_1_2_1_2_1_ | No | Neu5Ac |
| (37) | 7A5Q | Vc | 1.68 | P 2_1_2_1_2_1_ | No | Neu5Ac |
|  | 7A5C | Vc | 2.2 | P 6_3_ | Q54C-R1, R125A;L173C-R1 | Glycerol |
| (25) | 2WYK | Hi | 1.5 | P 2_1_2_1_2_1_ | No | Neu5Gc + SCN |
|  | 2V4C | Hi | 1.7 | P 2_1_2_1_2_1_ | No | KDN |
|  | 2WX9 | Hi | 1.37 | P 2_1_2_1_2_1_ | A11N | Neu5Gc |
|  | 2XA5 | Hi | 1.09 | P 2_1_2_1_2_1_ | A11N | Neu5Ac |
|  | 6H76 | Hi Rd KW20 | 1.50 (RT) | P 2_1_2_1_2_1_ | No | Neu5Ac, Cs, Cl |
|  | 6H75 | Hi Rd KW20 | 1.45 (RT) | P 2_1_2_1_2_1_ | A11N | Neu5Ac |
|  | 2WYP | Hi | 1.5 | P 2_1_2_1_2_1_ | A11N | KDN |
| (36) | 5LTC | Vc | 2.1 | P 2_1_2 2_1_ | Q54C-R1;R125A; L173C-R1 | -- |
| (26) | 2XWV | Hi | 1.05 | P 2_1_2_1_2_1_ | No | Neu5Ac |
|  | 2XWI | Hi | 2.195 | P 2_1_2_1_2_1_ | R147K | Neu5Ac |
|  | 2XWK | Hi | 1.49 | P 2_1_2_1_2_1_ | R147A | Neu5Ac |
|  | 2XWO | Hi | 1.54 | P 2_1_2_1_2_1_ | R147E | Sialylamide |
| (28) | 4MAG | Vc | 1.45 | I 2 2 2 | No | SO_4_, Co (II) |
|  | 4MMP | Pm | 1.57 | P 2_1_2_1_2_1_ | No | Neu5Ac |
|  | 4MNP | Fn | 2.5 | P 2_1_2_1_2_1_ | No | Neu5Ac |
| *PDB only* | 2XXK | Hi | 1.48 | P 2_1_2_1_2_1_ | N150D | Neu5Ac |
| (8) | 3B50 | Hi | 1.4 | P 2_1_2_1_2_1_ | No | Neu5Ac |
| (27) | 2CEY | Hi | 1.7 | I 2 2 2 | No | Zn |
|  | 2CEX | Hi | 2.2 | C 2 | No | Neu5Ac2En, glycerol, Zn |

**Supplementary Table 3 |** The root mean square difference (Å) between chains in the unbound *Aa*SiaP crystal asymmetric unit and the closed-bound structure, also compared with other SiaP homologues. Results show that the closed-unbound monomers of our structure (chains A and B) align best with the closed ligand-bound structure of *Aa*SiaP and homologous proteins *Pm*SiaP (75% sequence identity) and *Fn*SiaP (67% sequence identity).

|  |  | mostly closed-unbound | | open-unbound | | closed-bound |
| --- | --- | --- | --- | --- | --- | --- |
|  |  | chain A | chain B | chain C | chain D | + Neu5Ac |
| *Aa*SiaP | chain A mostly closed-unbound (9BH3) | - | 0.274 | 1.655 | 1.474 | 0.406 |
|  | chain B mostly closed-unbound (9BH3) | 0.274 | - | 1.620 | 1.541 | 0.285 |
|  | chain C open-unbound (9BH3) | 1.655 | 1.620 | - | 0.269 | 1.873 |
|  | chain D open-unbound (9BH3) | 1.474 | 1.541 | 0.269 | - | 1.722 |
|  | chain A + Neu5Ac (9BHF) | 0.406 | 0.285 | 1.873 | 1.722 | - |
|  |  |  |  |  |  |  |
| Other SiaP’s | *Hi*SiaP open-unbound (2CEY) | 1.759 | 1.683 | 0.471 | 0.469 | 1.876 |
|  | *Hi*SiaP closed-bound (6H76) | 0.515 | 0.415 | 2.097 | 1.914 | 0.440 |
|  | *Pm*SiaP closed-bound (4MMP) | 0.713 | 0.624 | 2.322 | 2.166 | 0.614 |
|  | *Fn*SiaP closed-bound (4MNP) | 0.765 | 0.630 | 2.004 | 1.843 | 0.640 |
|  | *Pp*SiaP closed-bound (7T3E) |  |  |  |  | 0.61 |
|  | *Vc*SiaP closed-bound (7A5Q) |  |  |  |  | 0.62 |
|  | *Vc*SiaP open-unbound (4MAG) | 2.530 | 2.521 | 1.221 | 1.308 | 2.599 |

**Supplementary Table 4 |** Cubic box length and total number of ions and water molecules in each system, and length of each simulation.

| **system** | **cubic box length (nm)** | **Na^+^** | **Cl^−^** | **TIP3P waters** | **simulation length (ns)** |
| --- | --- | --- | --- | --- | --- |
| Open state | 9.2364 | 1 | - | 25,066 | 500 |
| Fully-closed state | 9.1664 | 1 | - | 24,545 | 500 |
| Mostly closed state | 9.2084 | 1 | - | 24,876 | 500 |
| Neu5Ac-bound state | 8.7731 | 64 | 63 | 21,237 | 500 |
| Neu5Ac-bound state + H2O | 8.9359 | 69 | 67 | 22,503 | 500 |

# Supplementary Figures


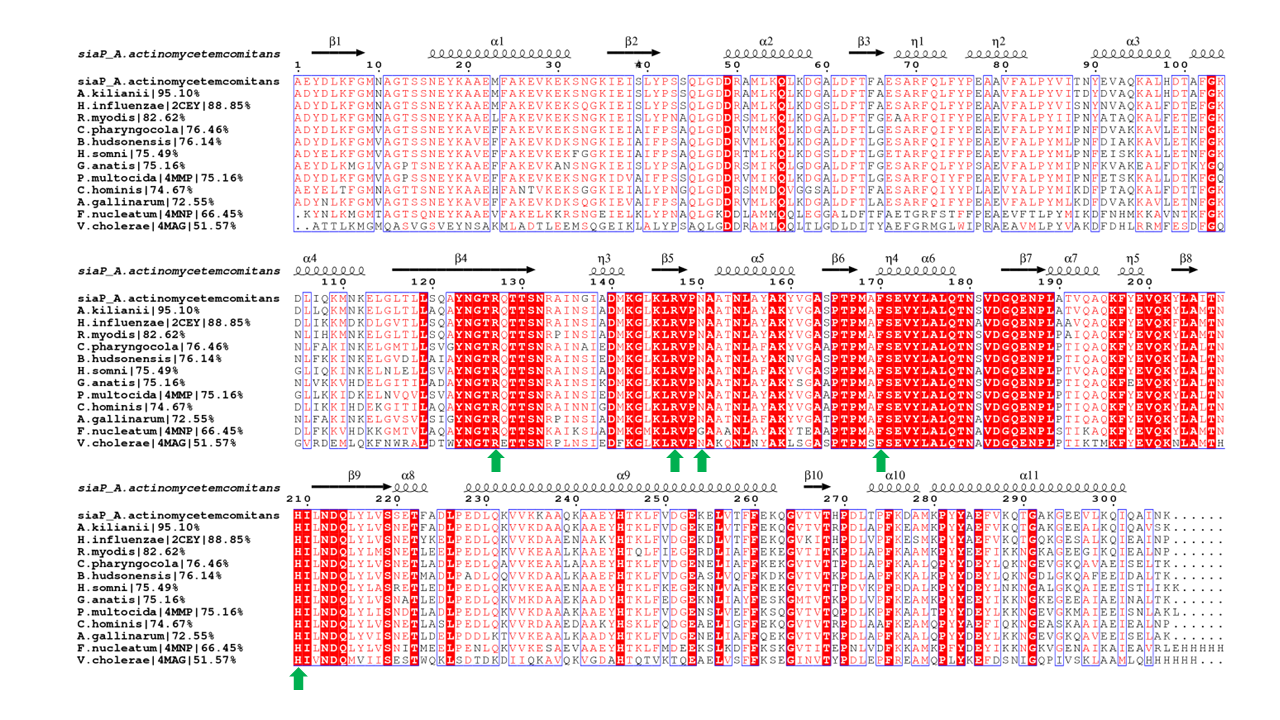


**Supplementary Figure 1 |** Multiple sequence alignment of 12 AaSiaP homologues (50–95% sequence identity), identified by PSI-BLAST using AaSiaP gene as the query term.


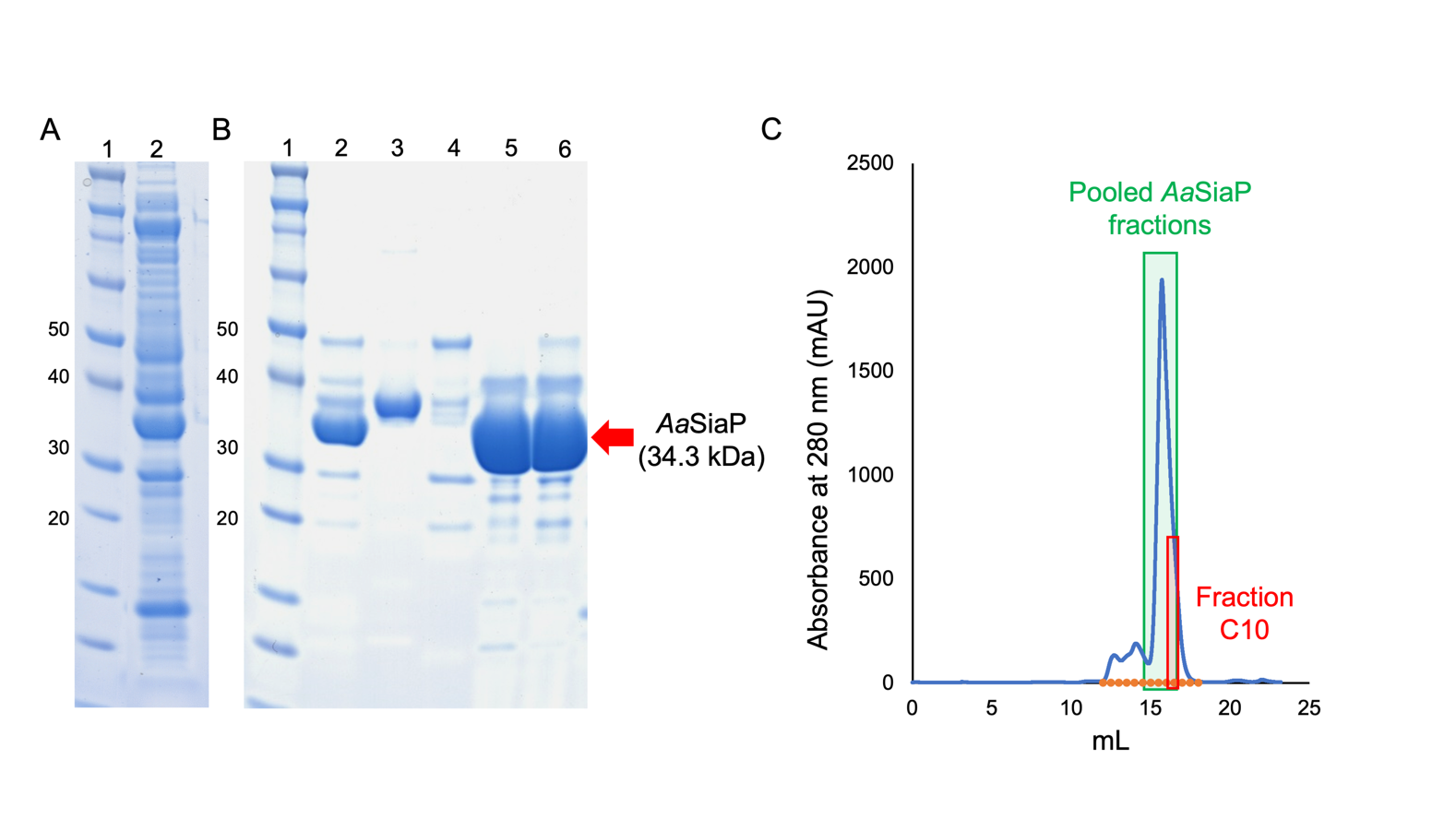
**Supplementary Figure 2 | A)** Recombinant *Aa*SiaP was highly produced and could be isolated from the periplasmic fraction. Lane 1: molecular-weight ladder, lane 2: crude periplasmic lysate. **B)** Recombinant *Aa*SiaP was then purified by anion-exchange chromatography, hydrophobic-interaction chromatography, and size-exclusion chromatography. Lane 1: molecular-weight ladder, lane 2: before size-exclusion chromatography, lanes 3/4: flowthrough fraction from size-exclusion chromatography, lane 5: post size-exclusion chromatography *Aa*SiaP containing-fraction C10 (red box in C), lane 6: post size-exclusion chromatography *Aa*SiaP showing pooled fractions from 15–16 mL (green box in C). **C)** *Aa*SiaP eluted from the SEC column as a single peak at approximately 15 mL, suggesting that it is a monodisperse species that remains stable throughout purification.


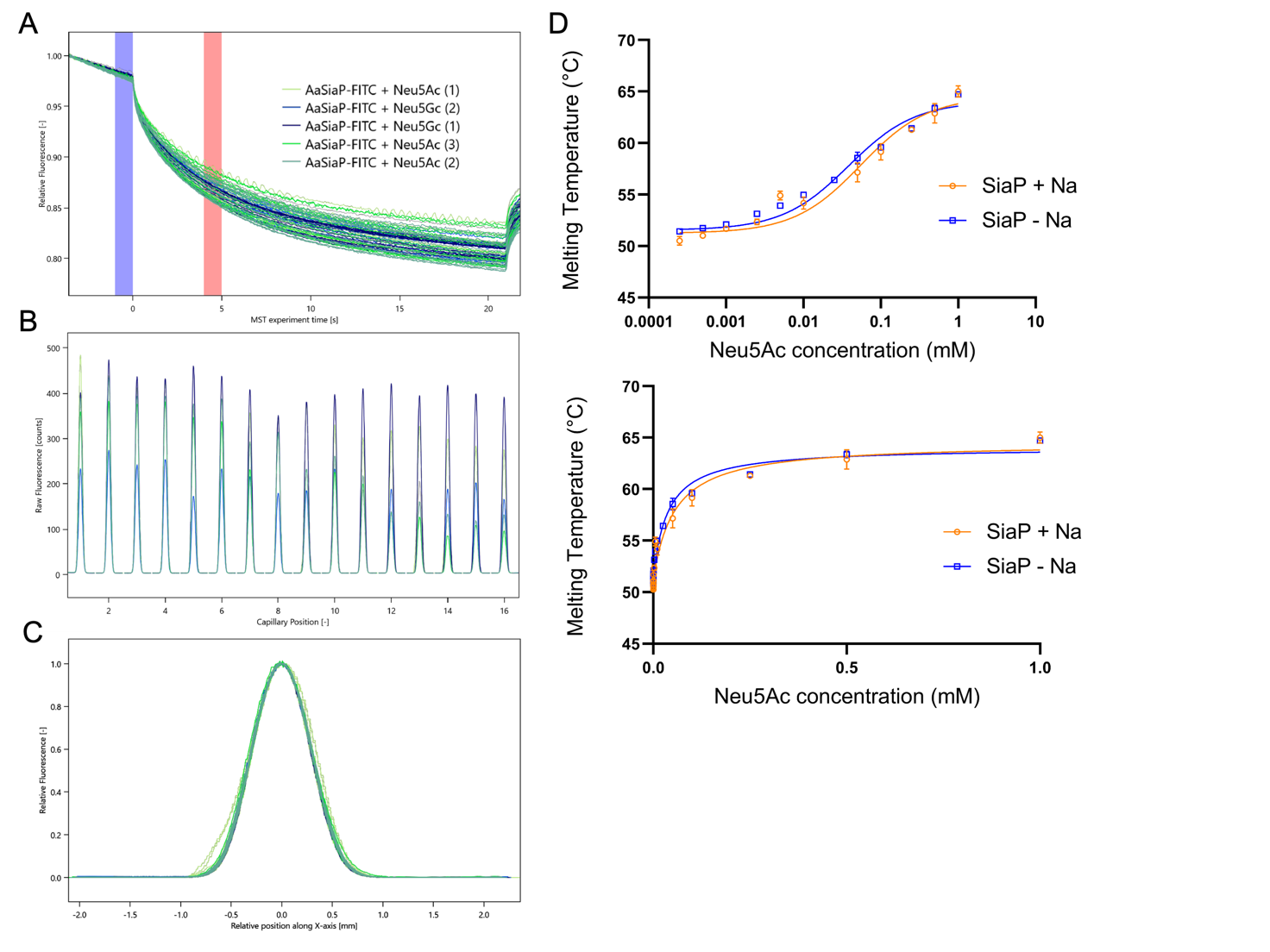
**Supplementary Figure 3 | A)** Raw MST traces for *Aa*SiaP-FITC with Neu5Ac (green curves) and Neu5Gc (blue curves), **B)** Capillary scans for MST, **C)** Capillary shape for MST data. **D)** Thermal shift assays with buffer containing-potassium salt instead of sodium had no effect on thermostability, suggesting that sodium is not required for binding. Titration data (as per Figure 2) plotted on a direct graph and fitted with a single-site ligand-binding model.


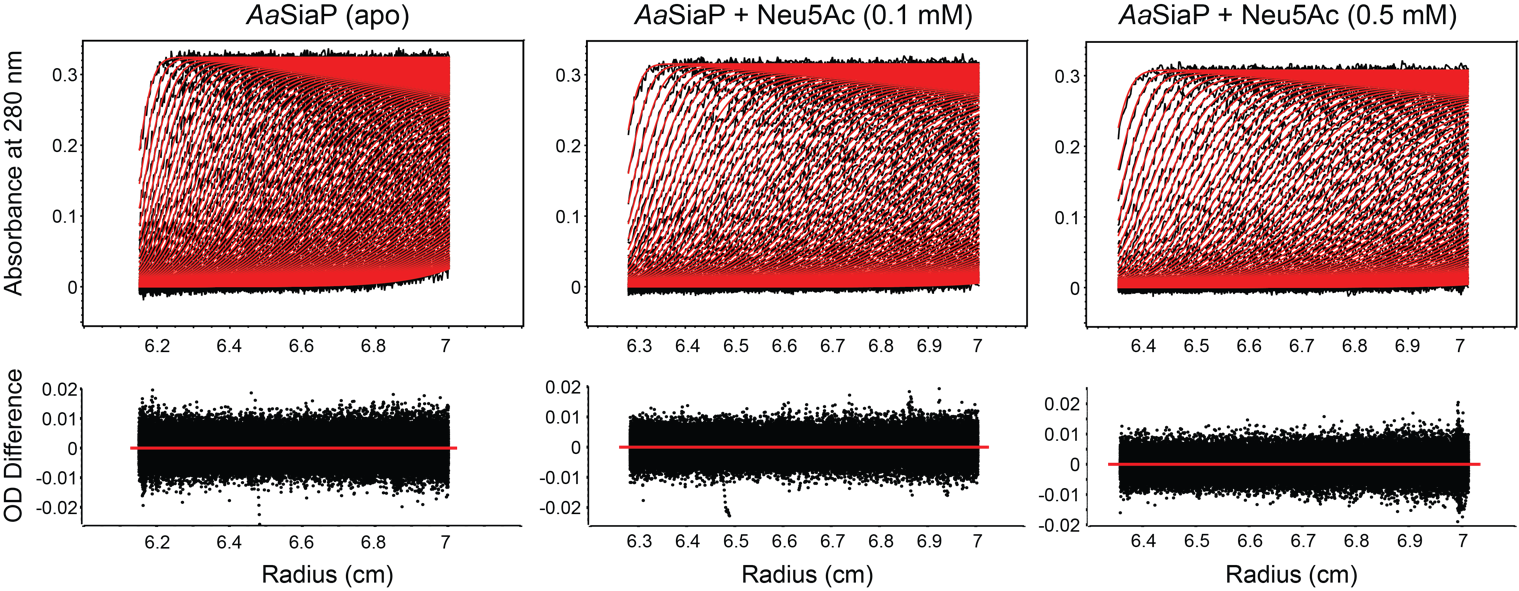
**Supplementary Figure 4 |** Sedimentation velocity data for *Aa*SiaP (black lines) and corresponding fit of the data to 2DSA-Monte-Carlo solutions and parsimonious regularisation by genetic algorithm analysis (red lines) for the analysis shown in **Figure 3B**. The residuals for the fit are also displayed (bottom).


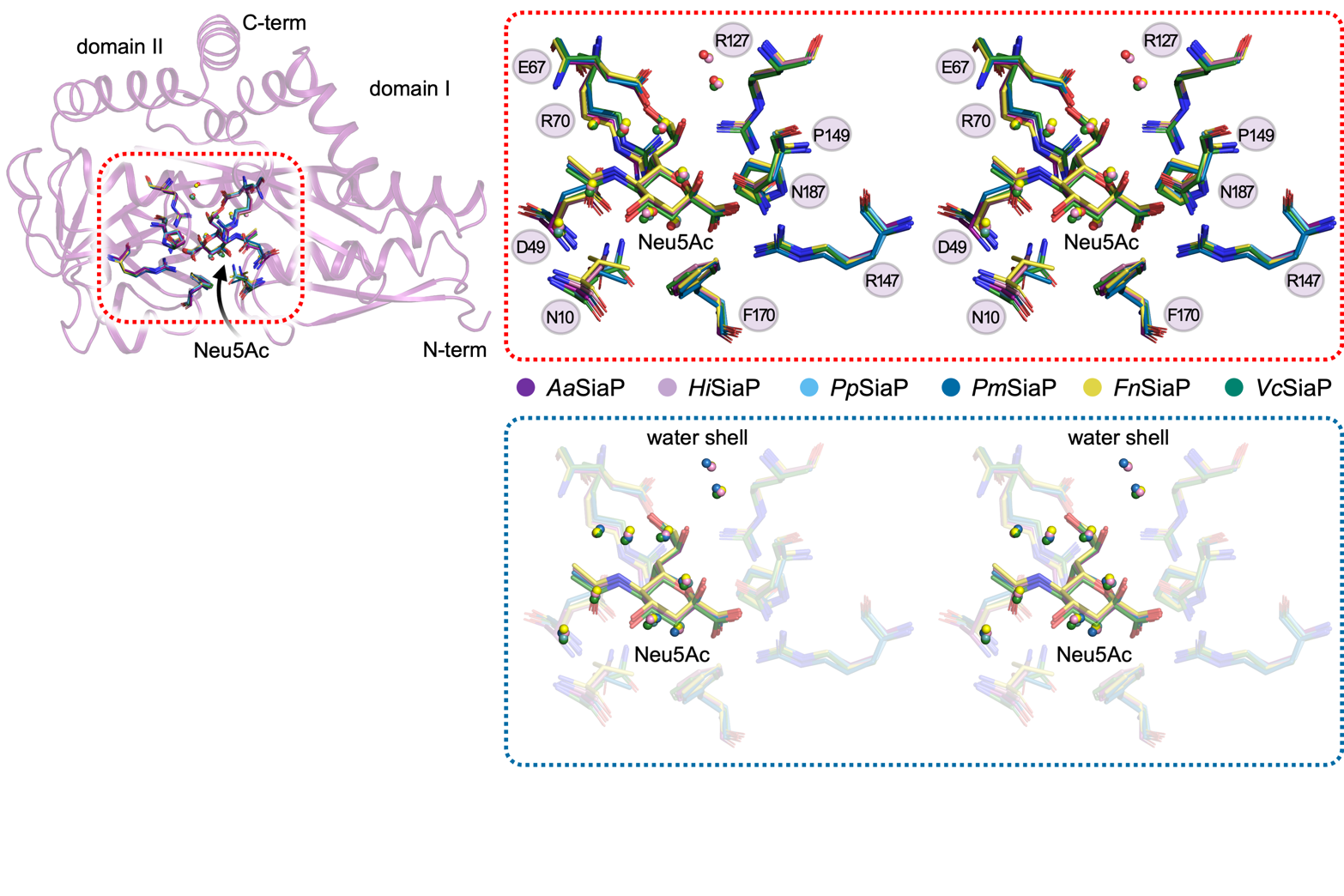
**Supplementary Figure 5 |** **Neu5Ac bound to *Aa*SiaP and overlay with homologues.** A focus on the Neu5Ac binding site demonstrates that both the contributing residues and the water shell around Neu5Ac is highly conserved. The top inset is a cross-eyed stereo plot of the binding site (red outline). The bottom inset (blue outline) is the same view, but highlighting the conserved water shell around Neu5Ac.

**
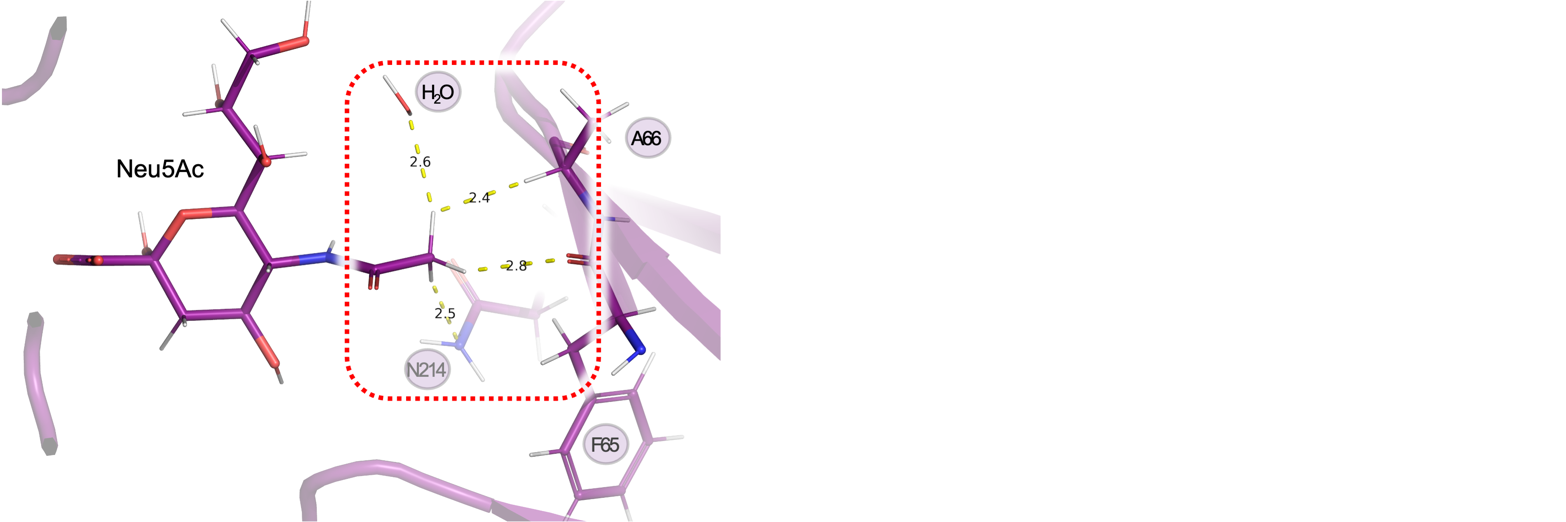
Supplementary Figure 6 |** **A focus on the binding interaction of the acetyl moiety of Neu5Ac.**

**
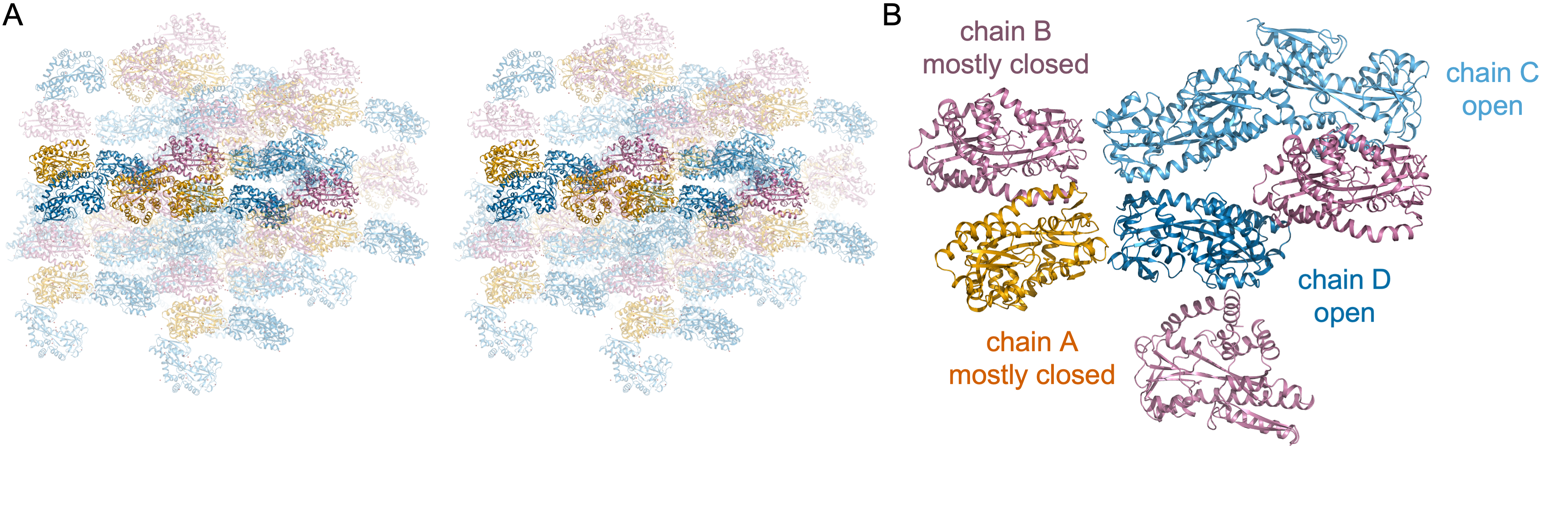
Supplementary Figure 7 |** **A stereo view of the crystal packing for the unbound-*Aa*SiaP structure.** The colouring of the monomers is the same as in **Figure 3A**; Chains A (orange) and B (magenta) are in a acetate-bound conformation, whereas chains C (light blue) and D (blue) are in an open conformation.
